# Gene expression profiles in Malpighian tubules of the vector leafhopper *Psammotettix striatus* (L.) revealed regional functional diversity and heterogeneity

**DOI:** 10.1186/s12864-022-08300-6

**Published:** 2022-01-21

**Authors:** Feimin Yuan, Cong Wei

**Affiliations:** Key Laboratory of Plant Protection Resources and Pest Management of the Ministry of Education, College of Plant Protection, Northwest A&F University, Yangling, 712100 Shaanxi China

**Keywords:** Cicadellidae, Osmoregulation, Organic solute transport, Detoxification, Immunity, Brochosome, Bioinformatics, RNA-Seq, RT-qPCR

## Abstract

**Background:**

Many leafhoppers are known as pests and disease vectors of economically important plants. Previous studies of the physiological functions of vector leafhoppers have mainly focused on the salivary glands and the alimentary tract that are deemed to be associated with digestion, host defense and phytoplasma and/or virus transmission. By contrast, the significance of Malpighian tubules (MTs) is less studied. To clarify the physiological function of MTs of the vector leafhopper *Psammotettix striatus* that transmits phytoplasma triggering the wheat blue dwarf disease, we performed a transcriptome study on *P. striatus* MTs and compared gene expression profiles among different anatomical regions in the tubules (i.e., MT1+2, the anterior segment together with the sub-anterior segment; MT3, the inflated segment; and MT4, the distal segment).

**Results:**

Transcriptome of *P. striatus* MTs generate a total of 42,815 high-quality unigenes, among which highly expressed unigenes are mainly involved in organic solute transport, detoxification and immunity in addition to osmoregulation. Region-specific comparative analyses reveal that all these MT regions have functions in osmoregulation, organic solute transport and detoxification, but each region targets different substrates. Differential expression and regional enrichment of immunity-related effector activities and molecules involved in phagocytosis and the biosynthesis of antimicrobial peptides among different regions indicate that MT1+2 and MT4 have the ability to eliminate the invading pathogens. However, in MT3 which secrets brochosomes to the integument and eggs as physical barriers, disulfide-isomerase, acidic ribosomal protein P and many other unigenes were highly expressed, which can be attractive candidate genes for future studies of the biosynthesis and the origin of brochosomes.

**Conclusions:**

*Psammotettix striatus* MTs perform multiple physiological functions as versatile organs than just excretory organs with osmoregulatory function. Heterogeneity of physiological functions among different MT regions is related to organic solute transport, detoxification, immunity and brochosome biosynthesis in addition to osmoregulation, and each region targets different substrates. These functions may be helpful for *P. striatus* to resist pathogens from habitats and to utilize a wider range of host plants, which may assist the transmission and spread of phytoplasmas. The results provide potential molecular targets for the exploit of chemical and/or gene-silencing insecticides.

**Supplementary Information:**

The online version contains supplementary material available at 10.1186/s12864-022-08300-6.

## Background

The Malpighian (renal) tubules (MTs) and hindgut of insects together constitute the function analogous to that of the mammalian kidneys [1]. Together with the rectum, MTs form the key excretory and osmoregulatory organs of insects [2, 3]. MTs play a crucial role in osmoregulation through the transepithelial transport of ions, water and other compounds from the hemolymph to the lumen during excretion. However, increasing evidence suggests the versatility of the physiological function of MTs than just water and ion transport. MTs express plenty of organic solute transporters, indicating the tubules have the capability to actively excrete the broadest range of organic solutes (e.g., sugar, amino acid and multivitamin) [4]. MTs express multiple detoxification-related genes, e.g., cytochrome P450 monooxygenases (P450s), glutathione *S*-transferases (GSTs), alcohol dehydrogenases (ADHs), uridine diphosphate-glycosyltransferases (UGTs) and ATP-binding cassette (ABC) transporters, suggesting that the tubules have physiological functions in the detoxification of endogenous secondary metabolites and xenobiotics (e.g., plant allelochemicals: ouabain, salicylate and vinblastine) or toxins (e.g., insecticides) [5–8]. MTs also express a variety of immunity-related genes involved in immunological responses, which sense pathogenic challenge (e.g., bacteria, fungi, viruses and protozoa) and mount effective killing responses by triggering the generation of effector activities (phagocytosis) and effector molecules (e.g., attacin, defensin and cecropin) [9]. Hence, MTs are considered to be completely independent immune tissue from the canonical immune tissue, the fat bodies [3, 10]. MTs have been also found to perform key functions in stress response, e.g., oxidative, desiccate and osmotic (salt/ionic) tolerance [9, 11–13]. Moreover, there are some specialized functions involved in MTs of some insects, such as producing silk [14], secreting protective material (such as brochosome) [15–18], storing mineralized granules [19, 20] and emitting bioluminescence [8, 21, 22]. In some insect species, MTs are also involved in carbohydrate transport and metabolism, development and reproduction [5, 23–26].

The phytophagous vector leafhopper *Psammotettix striatus* (L.) (Hemiptera: Cicadellidae: Deltocephalinae) is an economically important agricultural pest of winter wheat in northwestern China. It causes great yield losses of the crop through transmitting phytoplasma which triggers wheat blue dwarf (WBD) disease in a persistent circulative propagative manner [27]. Therefore, effective control of *P. striatus* plays an important role in the prevention and control of this disease. Previous studies have documented bionomics [28], development and fecundity [29], and morphology and ultrastructure of the mouthparts [30], antennae [31], digestive system as well as MTs [32] and spermatozoa [33] of *P. striatus*. However, the physiological functions of internal organs (including MTs) of this species have never been investigated.

**Fig. 1:**
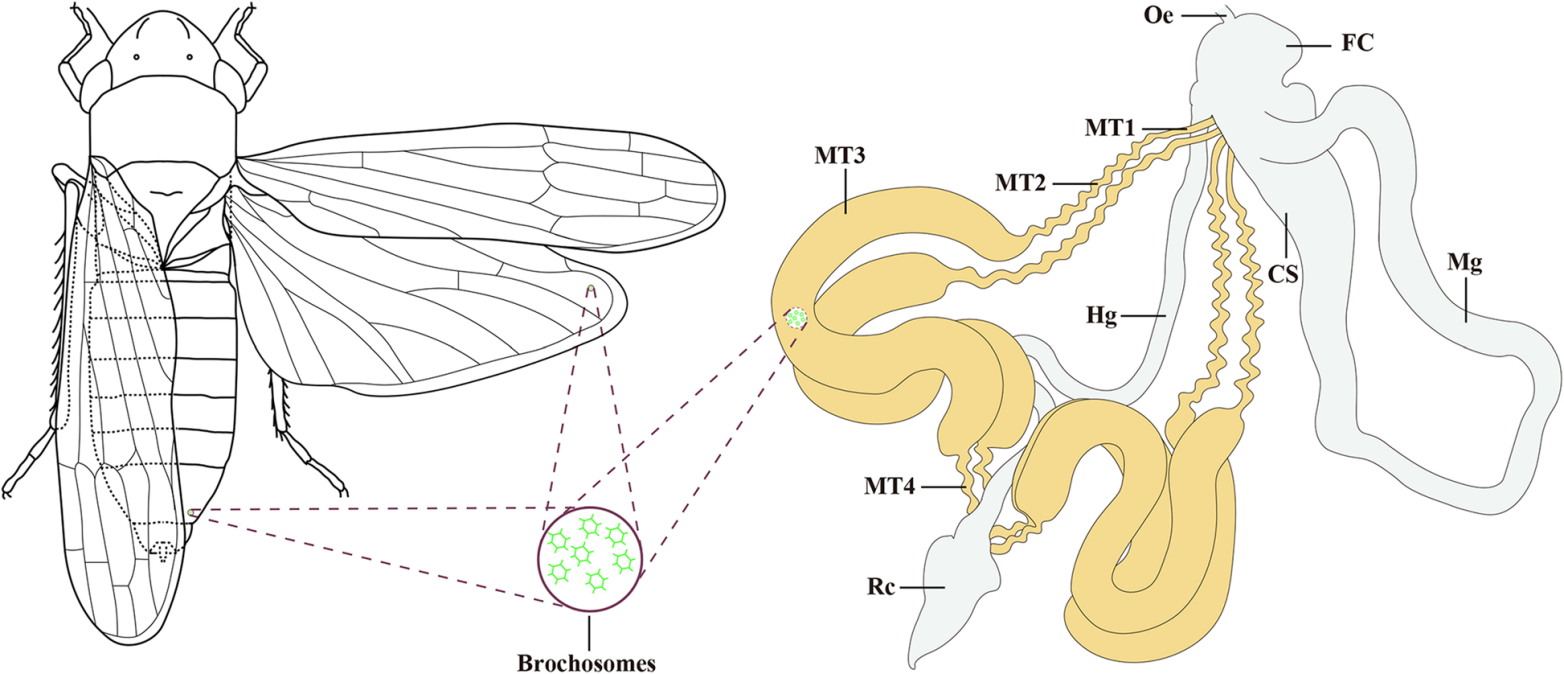
A schematic illustration of *P. striatus* and its brochosomes, alimentary tract and Malpighian tubules. CS, conical segment; FC, filter chamber; Hg, hindgut; Mg, midgut; MT1, the anterior segment of the Malpighian tubule; MT2, the sub-anterior segment of the Malpighian tubule; MT3, the inflated segment of the Malpighian tubule; MT4, the distal segment of the Malpighian tubule; Oe, oesophagus; Rc, rectum

There are four MTs in *P. striatus* and other leafhoppers, which emerge from the same side of the filter chamber at the interface between midgut and hindgut, and extend downward along the hindgut toward the dilated rectum [32, 34, 35]. Each tubule is formed by a single layer of epithelial cells around the lumen, which is segmented into four quite different segments with morphological and ultrastructural similarities and differences (Fig. 1). Each segment of MTs in leafhoppers shows relatively complex morphology and ultrastructure: the anterior segment (MT1) is slender and tubular, with epithelial cells containing numerous mitochondrial and lamellar rough endoplasmic reticulum; the sub-anterior segment (MT2) is wave-like in shape, with many vesicles and extensive rough endoplasmic reticulum in the cells; the inflated segment (MT3) is opaque and dilated tubular, containing a number of brochosome-containing vesicles in most of the cytoplasm of this region; and the distal segment (MT4) is undulate and appears to be transparent and lobulated, with cells containing a lot of mitochondria, vesicles and extensive rough endoplasmic reticulum. Numerous well-organized and extensive brush borders of microvilli exist in the epithelial cells of these four segments [32]. Physiological functions are likely to vary among the defined segments of MTs in leafhoppers concerning their respective cellular composition. Exploring the physiological functions and the molecular mechanisms underlying the functions of *P. striatus* MTs may be informative to uncover the morphological/functional differentiation of MTs in Membracoidea.

Herein, the RNA high-throughput sequencing technology was employed to characterize the functional diversity of different MT regions of *P. striatus*, i.e., MT1+2 (the anterior segment together with the sub-anterior segment), MT3 (the inflated segment), and MT4 (the distal segment). We aim to: (i) establish a de novo transcriptome of *P. striatus* MTs; (ii) test whether leafhopper MTs perform multiple physiological functions as versatile organs than just excretory organs with osmoregulatory function; (iii) describe putative region-specific molecular mechanisms of the MTs; and (iv) lay the foundation for future studies of the biosynthesis of brochosomes. These efforts will improve our understanding of physiological functions and molecular mechanisms underlying the functions of MTs in leafhoppers and treehoppers, and provide information for future studies of the biosynthesis of brochosomes and the immune responses in related pests.

## Results

### De novo assembly and annotation

We combined the nine cDNA libraries constructed from MT1+2, MT3 and MT4, respectively, to generate the complete assembly of *P. striatus* MTs. In total, 590 million raw reads containing 82.54 Gb of sequence data were obtained (all numbers associated with individual libraries can be seen in Additional file 1: Table S1). After filtering out adaptor sequences, poly-N and low-quality sequences, a total of 582 million clean and high-quality reads were remained, which accumulated to a total of 77.42 Gb. Results displayed that the GC content is about 52% in all the nine sequencing libraries, and the Q20 (the probability of an incorrect base call 1 in 100 times) of all libraries is above 97%, indicating that the transcriptome sequencing quality is sufficient for further analyses. Assembly of these clean reads yielded 42,815 high-quality unigenes ranging from 201 bp to 28,260 bp, with average length and N50 length being 867 bp and 1641 bp, respectively. The BUSCO (Benchmarking Universal Single-Copy Orthologs) analysis shows a level of 95.5% completeness for the assembly (83.13% complete and single-copy orthologs, and 12.37% complete and duplicated orthologs), indicating the high quality of the assembly completeness.

For functional annotation, 22,699 in 42,815 unigenes from the complete assembly of *P. striatus* MTs were matched to NCBI Non-redundant (Nr) protein database (http://www.ncbi.nlm.nih.gov), Swiss-Prot protein database (http://www.expasy.ch/sprot), Kyoto Encyclopedia of Genes and Genomes (KEGG) database (http://www.genome.jp/kegg), euKaryotic of Orthologous Groups of proteins (KOG) database (http://www.ncbi.nlm.nih.gov/KOG) and Gene Ontology (GO) database (http://www.geneontology.org/) using BLASTx search (E-value <1.0E–5, see Additional file 2: Fig. S1A). In total, 21,691 (50.66%) unigenes have significant matches in the Nr database, followed by 14,283 unigenes (33.36%) in the Swiss-Prot database, 18,166 unigenes (42.43%) in the KEGG database, 12,877 unigenes (30.08%) in the KOG database, and 3519 unigenes (8.22%) in GO database; while 20,116 unigenes show no significant hits in any of the databases. The results mentioned above suggest that MTs likely have some uncharacterized unigenes which may be novel genes that perform important functions in *P. striatus* MTs.

Based on E-values in the Nr database, more than half (53.4%) of the annotated unigenes have strong homology matches (Additional file 2: Fig. S1B). The highest percentage of unigenes were matched with the termite *Zootermopsis nevadensis* (10.05%), followed by the leafhopper *Graphocephala atropunctata* (7.19%), the whitefly *Bemisia tabaci* (6.75%), the brown marmorated stink bug *Halyomorpha halys* (6.16%), and the bed bug *Cimex lectularius* (5.73%). For the remaining 46.60% of unigenes, they were matched with loci in other insects. Moreover, in the Swiss-Prot database, hits were mainly attributed to *Homo sapiens*, *Mus musculus*, *Drosophila melanogaster*, *Rattus norvegicus* and *Bos taurus* (Additional file 2: Fig. S1C).

Due to the lack of genomic sequence information of *P. striatus*, functional annotation of the predicted unigenes was mainly referred to species whose genomic information was documented using GO, KEGG and KOG (Additional file 3: Fig. S2). In total, 16,063 non-redundant unigenes were assigned to GO term of the three major functional ontologies in biological process, cellular component and molecular function, which were distributed into 51 categories (Additional file 3: Fig. S2A). Using the KEGG annotation system to analyze biological pathways of unigenes that were active in *P. striatus* MTs resulted in 18,166 unigenes being successfully mapped to 143 pathways (the top 26 of which are depicted in Additional file 3: Fig. S2B). For the KOG database, 12,877 unigenes were classified into 25 functional categories (Additional file 3: Fig. S2C). The large number of assembled unigenes from *P. striatus* MTs annotated to these five databases suggest that a high quality of transcripts have been obtained, and the results provide basic information to illustrate the functions in *P. striatus* MTs.

**Fig. 2:**
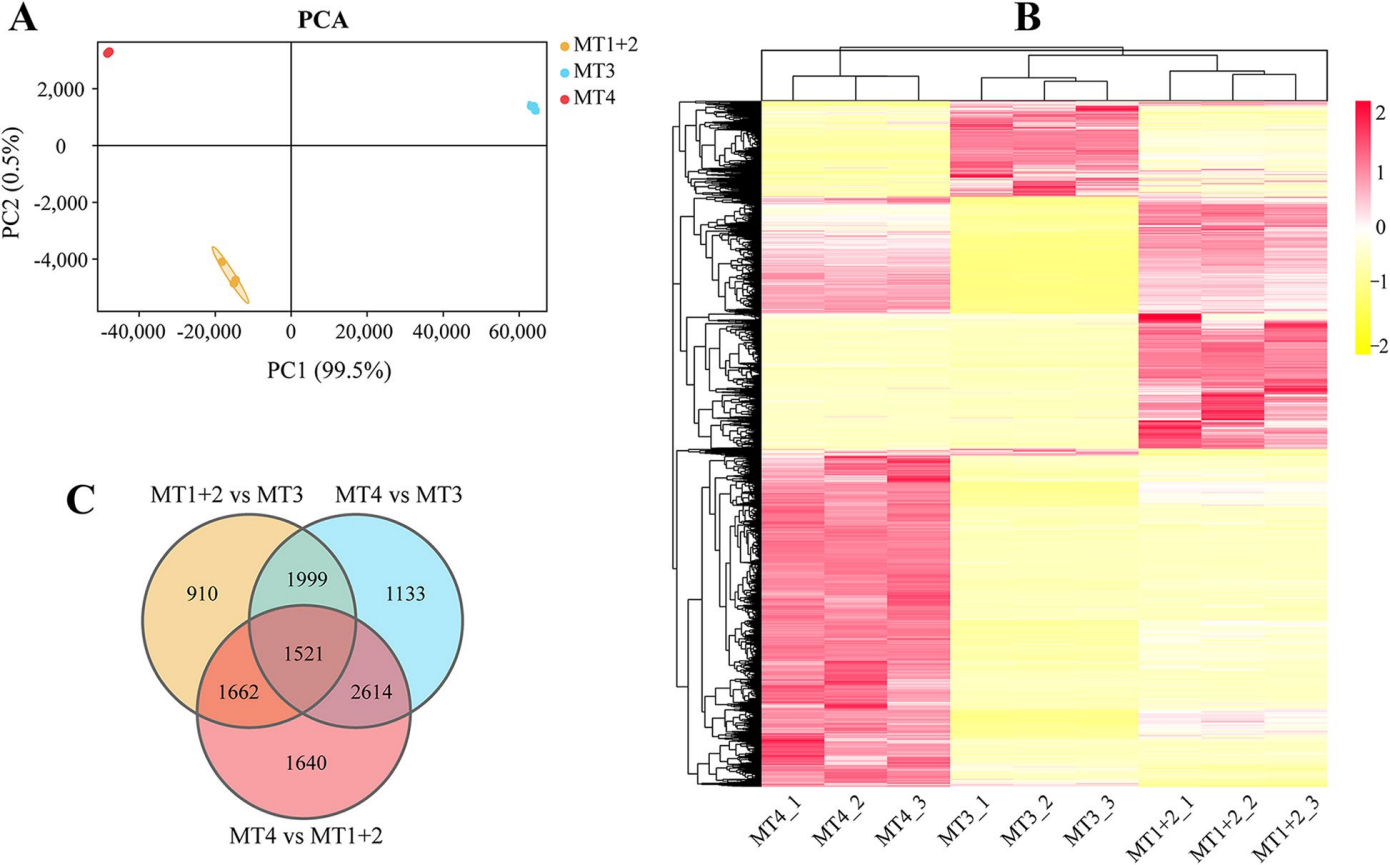
Gene expression pattern among different MT regions of *P. striatus*. **A** PCA for nine sequenced cDNA libraries, with the first two principal components (PC1 and PC2) based on transcriptomic results being shown. Each solid dot represents an individual cDNA library. **B** Hierarchical clustering heatmap of global gene expression based on normalized RPKM values from different regions. Red indicates higher expression, and yellow indicates lower expression. **C** Venn diagram exhibiting the number of common and unique unigenes between pairwise comparisons

The results of principal component analysis (PCA) on nine cDNA libraries show that all different MT regions are separated and all biological duplicates of the same region are clustered together (Fig. 2A), which coupled with the results of the Pearson correlation coefficient between replicates in each region (>0.998) indicate high reliability of data and consistency between replicates (Additional file 4: Fig. S3). The results mentioned above indicate that the transcriptome data involved in this study are of high quality and sufficient for subsequent downstream bioinformatics analysis.

All sequencing data have been deposited in the NCBI Short Read Archive (SRA) database (accession number: SRP326705) affiliated with the BioProject PRJNA743281.

### The putative function of *P. striatus* MTs

In general, MTs are considered to play a crucial role in water removal and osmotic regulation during excretion. However, the hierarchical clustering heatmap shows a significantly disparate global gene expression pattern among different regions (Fig. 2B), suggesting that there may be differences in physiological functions of these regions. To reveal the potential physiological function of different regions in *P. striatus* MTs, the most abundant top 100 unigenes in MT1+2, MT3 and MT4 are displayed based on the RPKM (Reads Per Kilobase per Million mapped reads) values, respectively (Additional file 5: Table S2). The list indicates that *P. striatus* MTs are likely also involved in organic solute transport, detoxification and immunity in addition to osmoregulation. This emphasizes that MTs function not only as excretory organs with osmoregulatory function but also as defense tissues against the endogenous secondary metabolites (e.g., hydrogen peroxide) produced during the normal metabolic process, the ingested xenobiotics/toxins (e.g., plant allelochemicals and insecticides), and the encountered pathogens.

Furthermore, there are several highly enriched unigenes putatively involved in “lipid, amino acid and carbohydrate transport and metabolism” and “translation and processing of proteins” (Additional file 5: Table S2). Some unigenes, such as Unigene 0000394, Unigene 0027792, Unigene 0000862 and Unigene 0000693, are highly abundant among all the MT regions of *P. striatus*, but they have not been annotated and classified. Moreover, a relatively substantial fraction of unigenes at the top of the list based on RPKM values are novel and “unknown” (shown no similarities with the genes deposited in the abovementioned five databases), which may play vital roles in basic tubular physiological function and/or may be expressed as species- and/or MTs-specific genes. For instance, of the top 100 unigenes in MT3 (the inflated segment which has the ability to produce brochosomes) nearly a third (31.0%) have not been annotated, and only 10% have been estimated of function. These unigenes with no functional annotation and classification in MT3 are likely to be closely related to the synthesis of brochosome.

### Differentially expressed genes (DEGs) and enrichment analysis

**Fig. 3:**
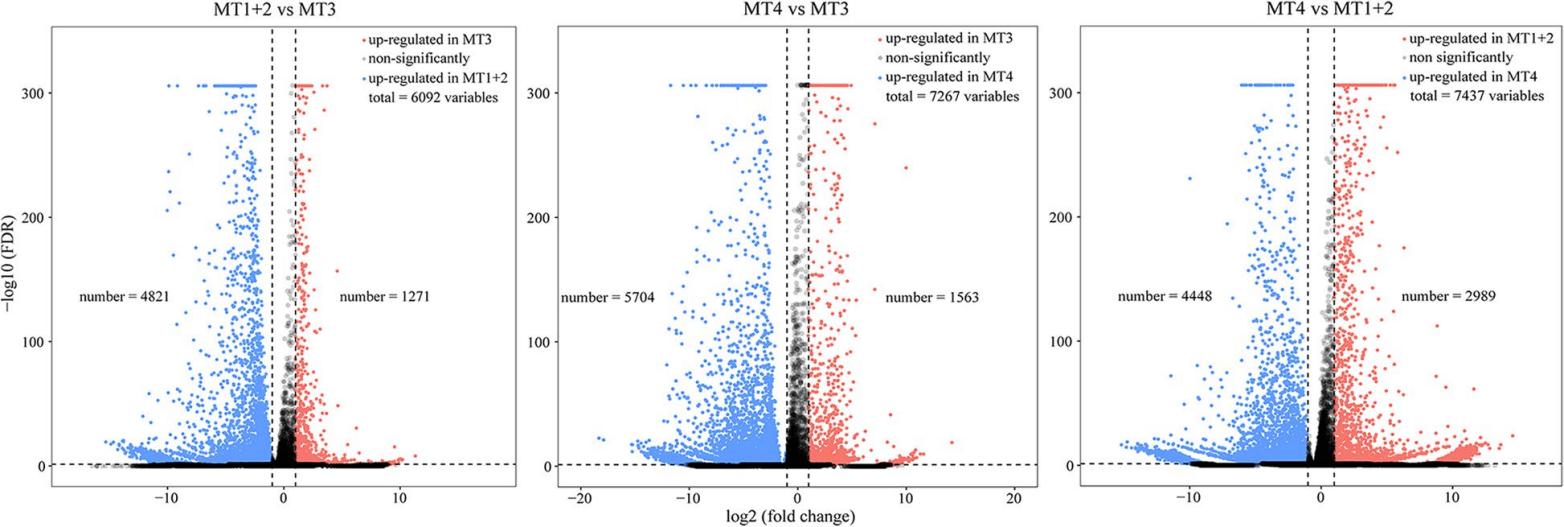
Volcano plots demonstrating the DEGs of all detected genes from the RNA-Seq dataset among different MT regions of *P. striatus*. DEGs are colored blue and red

The number of pairwise comparative DEGs among different regions is summarized in a venn diagram (Fig. 2C). Volcano plots show that the strongest gene expression differences are in the pairwise comparison between MT4 and MT1+2 with 7437 DEGs (4448 up-regulated in MT4 and 2989 up-regulated in MT1+2), followed by the comparison between MT4 and MT3 with 7267 DEGs (5704 up-regulated in MT4 and 1563 up-regulated in MT3), and between MT1+2 and MT3 with 6092 DEGs (4821 up-regulated in MT1+2 and 1271 up-regulated in MT3), respectively (Fig. 3).

KEGG expression enrichment was analyzed for all the significant DEGs. In the comparison of MT1+2 versus MT3, DEGs were most highly enriched in oxidative phosphorylation, drug metabolism-other enzymes, citrate cycle (TCA cycle), glutathione metabolism, and peroxisome. In the comparison of MT4 versus MT3, DEGs were most highly enriched in oxidative phosphorylation, Toll and Imd signaling pathway, lysosome, propanoate metabolism, and TCA cycle. In the comparison of MT4 versus MT1+2, DEGs were most highly enriched in drug metabolism-other enzymes, drug metabolism-cytochrome P450, metabolism of xenobiotics by cytochrome P450, glutathione metabolism, and neuroactive ligand-receptor interaction (the top 20 in each pairwise comparisons are depicted respectively in the Additional file 6: Fig. S4). Through the KEGG enrichment analysis, we got that DEGs are mainly related to detoxification, immunity and energy supply, indicating the difference in physiological function among different MT regions. In addition, the *p*-value of the KEGG enrichment indicates that the largest number and highest enriched degree of DEGs are involved in oxidative phosphorylation, showing that different MT regions have different demands on energy in the execution of the normal physiological function.

GO term enrichment analyses were also performed for all the significant DEGs. Among the pairwise comparisons, DEGs were highly enriched in biological processes including localization, oxidation-reduction process, protein complex assembly, ion homeostasis, and transport. DEGs were highly enriched in molecular functions including transport activity, oxidoreductase activity, transaminase activity, and channel activity. DEGs were highly enriched in cellular components including proton-transporting two-sector ATPase complex, membrane, envelope, macromolecular complex, and ribonucleoprotein complex (the top 10 in biological processes, top 7 in molecular functions and top 3 in cellular components in the pairwise comparisons are depicted respectively in Additional file 7: Fig. S5). These GO terms mentioned above are mainly related to osmoregulation and organic solute transport, which also indicate the difference in physiological function among different MT regions.

Based on the above analyses, DEGs identified among different MT regions of *P. striatus* are mainly linked to osmoregulation, organic solute transport, detoxification, and immunity.

### Functional characterization of regionally enriched genes

Enrichment analysis of DEGs demonstrates ample unigenes with a preference for certain MT regions, suggesting that the annotations of these unigenes might reflect functional specializations of corresponding regions. To better survey the molecular basis mainly of osmoregulation, organic solute transport, detoxification and immunity in the tubules, we perform a quantitative comparison among different MT regions of *P. striatus* based on RPKM values from RNA-Seq data.

### Osmoregulation and organic solute transport

**Fig. 4:**
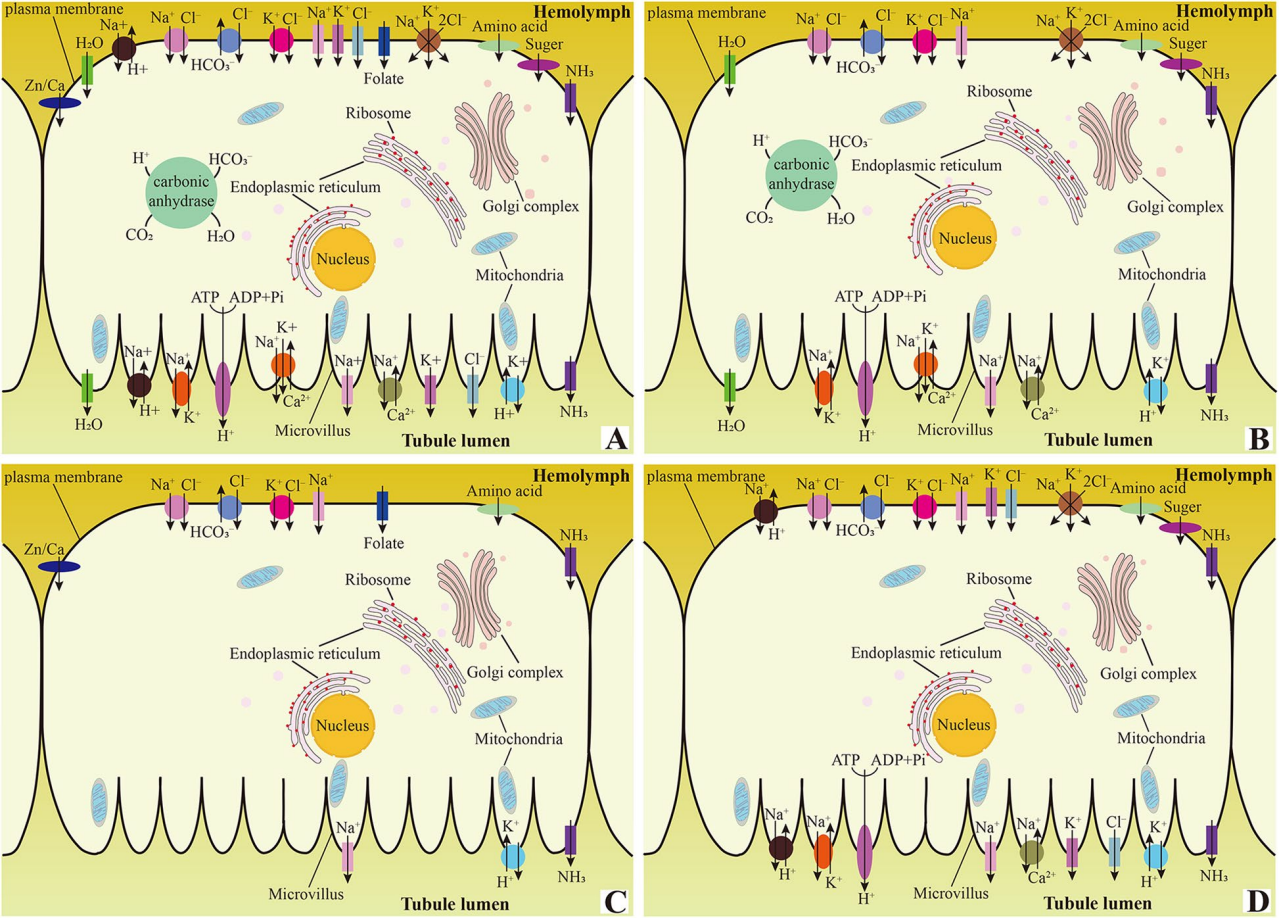
Schematic illustrations of molecules associated with transepithelial fluid transport in *P. striatus* MTs. **A** General catalog. **B** Transporters associated with transepithelial fluid transport in a cell of MT1+2. **C** Transporters associated with transepithelial fluid transport in a cell of MT3. **D** Transporters associated with transepithelial fluid transport in a cell of MT4

Here, we identified 28 DEGs encoding proteins involved in osmoregulation and organic solute transport among MT1+2, MT3 and MT4 (Fig. 4; Additional file 8: Table S3; Additional file 9: Fig. S6). The proteins encoded by these DEGs include aquaporin (AQP), ion-motive ATPases, ion channels, ion exchangers (antiporters), ion cotransporters (symporters), metal transporters, amino acid transporters, sugar transporters, vitamin transporters, NH_3_ transporter and carbonic anhydrase (Fig. 4A), which exhibit regional heterogeneity in their expression (Additional file 9: Fig. S6). MT1+2 demonstrates high levels of expression of AQP, carbonic anhydrase, Na^+^/K^+^/Ca^2+^ exchanger, Na+/nucleoside cotransporter, long-chain fatty acid cotransporter and solute carrier organic anion transporter, as well as sweet sugar transporter and facilitated trehalose transporter (Fig. 4B; Additional file 9: Fig. S6). MT3 shows relatively higher levels of proton-coupled folate transporter, zinc transporter, organic cation transporter, proton-coupled amino acid transporter, plasma membrane calcium-transporting ATPase, peptide transporter, and b (0, +)-type amino acid transporter (Fig. 4C; Additional file 9: Fig. S6). Lastly, MT4 expresses relatively higher levels of the inward-rectifying potassium (Kir) channel, chloride channel and sodium/hydrogen exchanger, as well as sodium/bile acid cotransporter, neutral and basic amino acid transporter rBAT-like, and also expresses excitatory amino acid transporter, V-ATPase, neutral and basic amino acid transporter rBAT, bumetanide-sensitive sodium-(potassium)-chloride cotransporter, sodium/potassium-transporting ATPase and facilitated glucose transporter at levels similar to those in MT1+2 (Fig. 4B, D; Additional file 9: Fig. S6). Additionally, MT4 demonstrates high levels of perprotachykinin and sodium/calcium exchanger (Fig. 4D; Additional file 9: Fig. S6). The differential expression of the abovementioned proteins related to transepithelial fluid transport suggests that different MT regions selectively transport distant substrates in the process of osmoregulation and organic solute transport, i.e., different MT regions showing heterogeneity of physiological functions.

### Detoxification

**Fig. 5:**
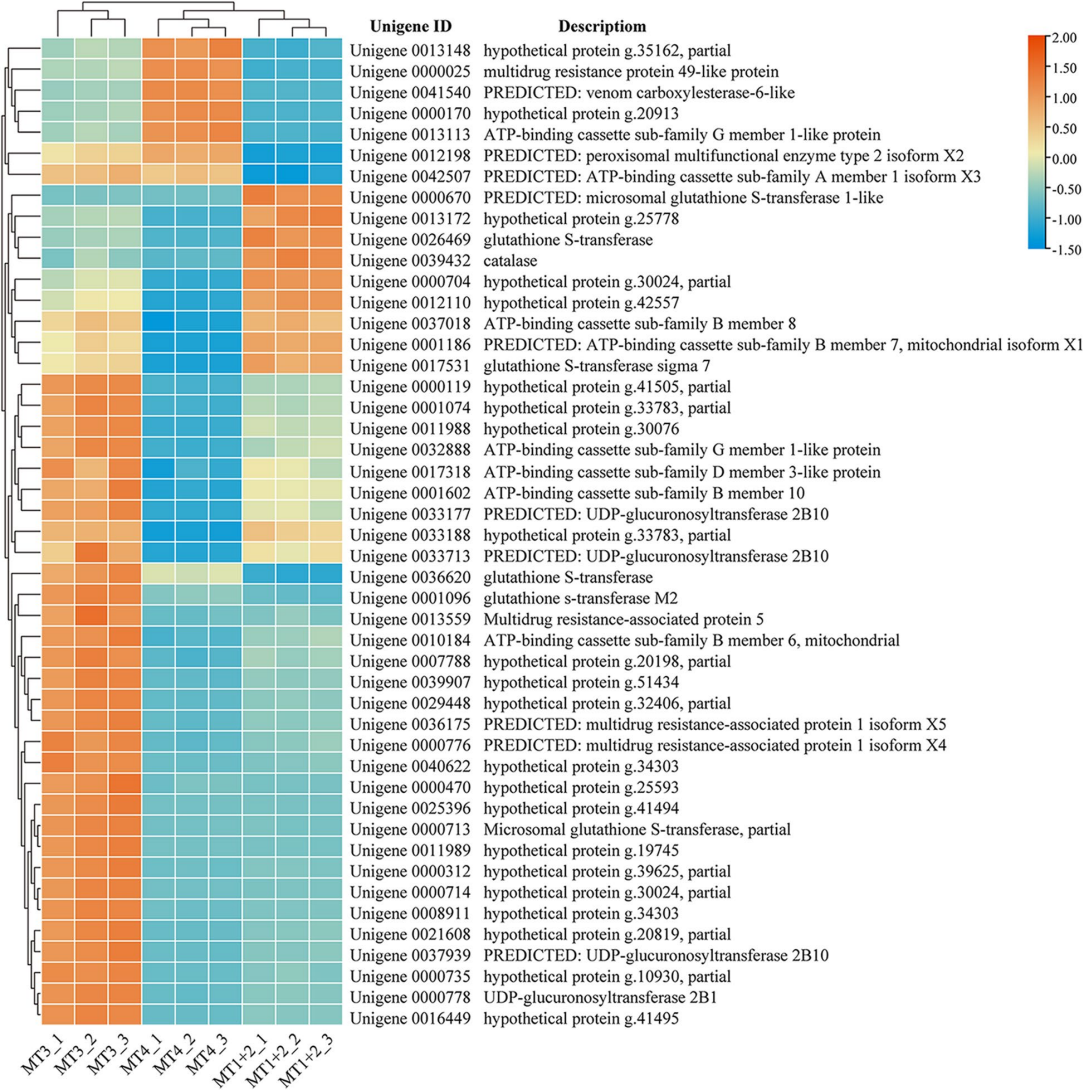
Normalized heatmap based on RPKM values of DEGs related to detoxification of *P. striatus* MTs. Orange color indicates up-regulated expression, whereas blue color indicates down-regulated expression

Forty-seven DEGs involved in detoxification were identified among different MT regions of *P. striatus*, including 12 ABC transporters (containing four multidrug resistance proteins (MRPs)), six GSTs, four UGTs, one catalase, one peroxisomal multifunction enzyme, one venom carboxylesterase, and 22 hypothesized proteins involved in endogenous secondary metabolites and xenobiotics biodegradation and metabolism (Fig. 5; Additional file 10: Table S4). Among which, most detoxification-related unigenes were exclusively highly expressed in MT1+2 when compared with MT3 and MT4, including seven ABC transporters, three GSTs, four UGTs and 16 hypothesized proteins. In addition, one ABC transporter, one MRP, one venom carboxylesterase and two hypothesized proteins were exclusively highly expressed in MT3. Two GSTs, one catalase and three hypothesized proteins were exclusively highly expressed in MT4. Furthermore, one peroxisomal multifunction enzyme and one ABC transporter expressed in MT3 are similar at levels to those in MT1+2. Likewise, two ABC transporters, one GST and one hypothetical protein expressed in MT4 are similar at levels to those in MT1+2. Gradients in the expression of detoxification-related unigenes in different MT regions of *P. striatus* argue that the three regions are specialized for targeting distant substrates, which confirm the heterogeneity of physiological function of MT regions in terms of detoxification of endogenous secondary metabolites and xenobiotics/toxins.

### Immunity

Through the transcriptome-based comparative analysis from different MT regions of *P. striatus*, we identified 43 DEGs related to immune responses (Additional file 11: Table S5; Additional file 12: Fig. S7). Of the immunity-related unigenes, 6, 1, 1 and 8 unigenes fell into recognition, modulation, signaling and effector, respectively. Among the unigenes involved in recognition, one peptidoglycan recognition protein (PGRP) was relatively highly expressed in MT1+2; one integrin and one PGRP were at similar expression levels between MT1+2 and MT4, whereas two *β*-glucan recognition proteins (GRP) and one scavenger receptor (SR) were relatively highly expressed in MT4. Of the unigenes related to modulation, serine protease (SP) was significantly highly expressed in MT4. Among those unigenes involved in the signaling pathway, spaetzle was significantly highly expressed in MT4. Of those unigenes related to effector molecules, eight antimicrobial peptides (AMPs) related to humoral immunity including one defensin and two superoxide dismutases (SODs) were highly expressed in MT1+2, and two defensins, two peroxiredoxins (Prx) and one SOD were at similar expression levels between MT1+2 and MT4. In addition, a variety of lysosomal-related acid hydrolases (e.g., acid phosphatase, cysteine proteinase and asparaginase, etc.) involved in cellular immunity were differentially expressed in different MT regions of *P. striatus* (Additional file 12: Fig. S7). But interestingly, our results show that all the differentially expressed unigenes related to immunity (including humoral immunity and cellular immunity) in MT3, the inflated segment, exhibited extremely lower levels when compared with MT1+2 and MT4 (Additional file 12: Fig. S7). Differentially expressed immunity-related unigenes uncover the discrepancy of the molecular mechanism of different MT regions in *P. striatus* in resisting encountered pathogens.

### Validation of RNA-Seq data by reverse transcription-quantitative PCR (RT-qPCR)

**Fig. 6:**
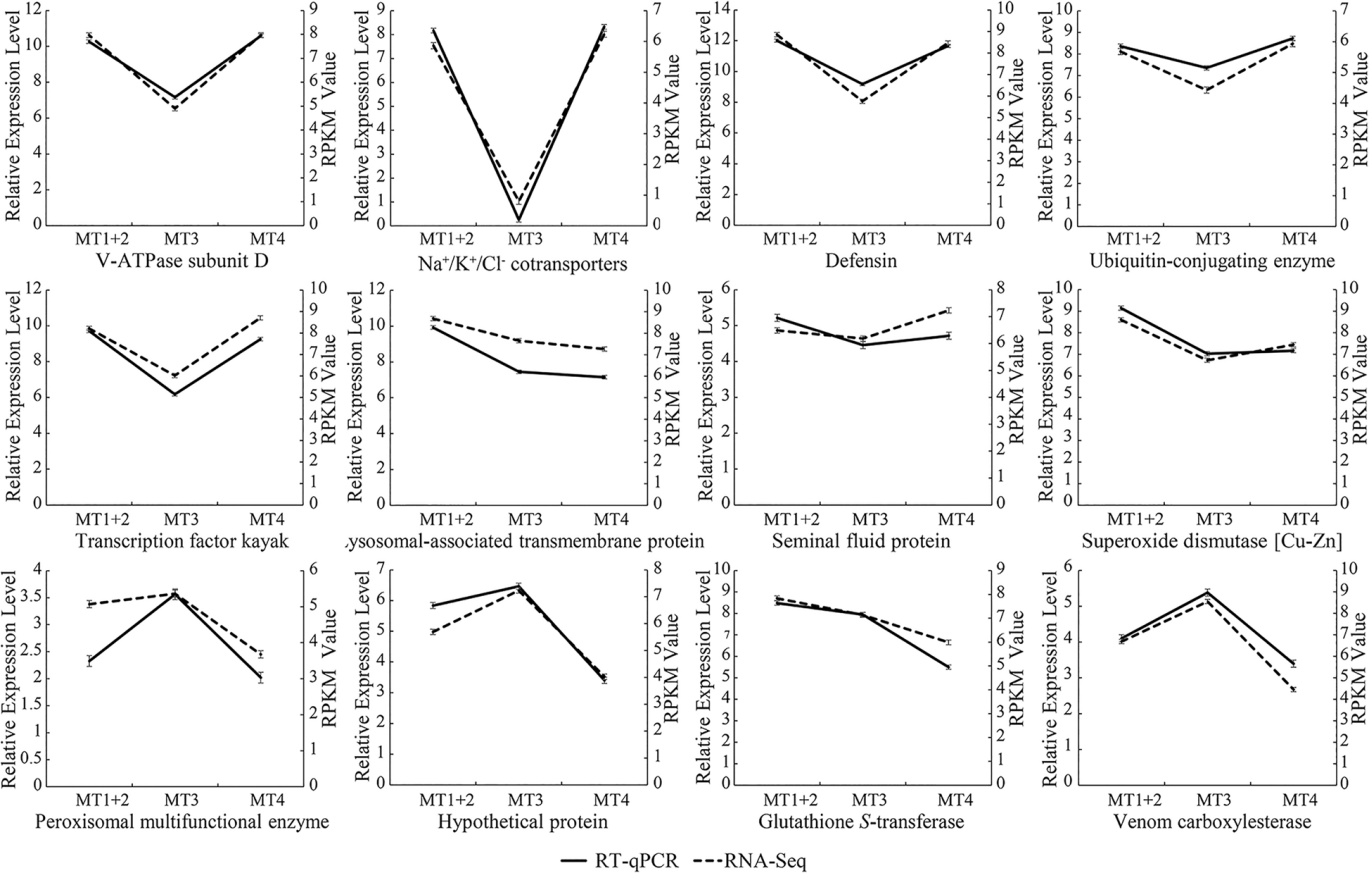
Gene expression levels of DEGs among different MT regions of *P. striatus* through RNA-Seq and RT-qPCR analyses

To verify the accuracy and reliability of RNA-Seq, and to further confirm the identified DEGs among different MT regions of *P. striatus*, the relative expression levels of 12 representative DEGs from enriching GO annotations and KEGG pathways were analyzed by RT-qPCR. These genes were selected because we are interested in their functions involved in osmoregulation, detoxification and immunity. The selected genes are as follows: V-ATPase subunit D (Unigene 0000156), Na^+^/K^+^/Cl^−^ cotransporter (Unigene 0001060), glutathione *S*-transferase (Unigene 0036620), venom carboxylesterase (Unigene 0041540), peroxisomal multifunctional enzyme (Unigene 0012198), hypothetical protein (Unigene 0000170), seminal fluid protein (Unigene 0009863), lysosomal-associated transmembrane protein (Unigene 0003385), superoxide dismutase (Unigene 0042801), transcription factor kayak (Unigene 0002827), ubiquitin-conjugating enzyme (Unigene 0017102), and defensin (Unigene 0001753) (All the primers for RT-qPCR used in this study are listed in Additional file 13: Table S6, and their specificity have been confirmed using NCBI Primer-BLAST). For each DEG, the relative expression levels performed by RT-qPCR are different among the three regions, which are the same with the results based on RPKM of RNA-Seq analysis (Fig. 6). Therefore, we conclude that the results of RNA-Seq and identified DEGs in this study are accurate and credible.

## Discussion

Plant diseases (e.g., Australian grapevine yellows disease, aster yellows disease and WBD disease) caused by phytoplasmas transmitted primarily by insect vectors such leafhoppers, planthoppers and psyllids [36] can result in minor to extensive damage in hundreds of commercial and native plants (e.g., grapevine, lettuce and wheat) [27, 37, 38]. More than 75% of all confirmed phytoplasma vector species come from the subfamily Deltocephalinae of the family Cicadellidae [36]. Transcriptome-based tissue-specific comparative analysis is a powerful research technique since patterns of gene expression are likely to vary among disparate tissues, organs, or even different regions of the same organ concerning their respective functions [39]. Correlations between the transcriptomes in many tissues or organs of animals (e.g., antennae, salivary glands, midgut, midgut ‘appendices’, hepatopancreas, venom gland, gill) and its respective physiological functions have been reported [40–45]. Similar studies have been carried out for the MTs of a few insect species, but these studies have been limited in viewing the MTs as a whole organ [3, 5, 10, 12, 22, 25, 46–50]. To our knowledge, studies of the transcriptome of vector leafhoppers have mainly focused on the salivary glands and the alimentary tract [51–55]. Physiological functions of MTs of vector leafhoppers have seldom been studied using transcriptome-based tissue-specific comparative analysis, which is partly due to the difficulty of dissecting and obtaining sufficient tissue for transcriptome sequencing from individuals of tiny size.

Insect body is anatomically simpler than that of mammals, thus tasks performed by certain organs in mammals must be shared out among tissues in insects [4]. Conversely, a single organ of insects may perform multiple physiological functions, which are in line with the transcriptome results of *P. striatus* MTs that the tubules with multiple physiological functions as versatile organs. In the present transcriptomic analysis of *P. striatus* MTs, the results of PCA on nine cDNA libraries, Pearson correlation coefficient between replicates and gene expression profile of all samples indicate that the global gene expression patterns are highly conserved within a specific MT region but quite variable among different regions (Fig. 2A, B; Additional file 4: Fig. S3). The discrepancy of gene expression patterns among different MT regions of *P. striatus* indicates the functional differences, since the area-specific molecules confer functional specificity. But as far as we know, no study has been able to split the specialized functions of the different MT regions based simply on genes that show enrichment within that region. When we identified and examined such genes, we analyzed the annotations of those with a striking resolution that reflected known functional specializations of the regions in question. Moreover, we examined novel functional relationships between different regions and their corresponding transcriptomes, respectively. The expression of the genes involved in “organic solute transport”, “detoxification”, “immunity”, “lipid, amino acid and carbohydrate transport and metabolism” and “translation and processing of proteins” in addition to osmoregulation in the tubules emphasizes that MTs not only as excretory organs with osmoregulatory function, but also as defense tissues against the endogenous secondary metabolites, the ingested xenobiotics/toxins, and the encountered pathogens, etc.

### Osmoregulation and organic solute transport in MTs

Insect MTs serve as the main excretory organs that generate primary urine to excrete excess ions or nitrogenous waste in the form of uric acid from the metabolism of nucleic acids and proteins [8], and as osmoregulatory organs that actively secrete transepithelial fluid and selectively reabsorb ions, water and desired solutes as the fluid passes through the tubule [26]. It is mediated by the cooperation of V-type H^+^ ATPase, AQPs, ion-motive ATPases, ion transporters, ion channels, ion exchangers and ion cotransporters, etc. [1], and regulated by neuropeptides [56]. Osmoregulation and organic solute transport during excretion performed by MTs are essential for the survival of insects in a wide range of habitats and diets. Insects rapidly and effectively excrete excess ingested water and ions, and concentrate the nutrient-rich food to reduce the volume of inflated hemolymph after a feeding, so that they are more mobile and less susceptible to predation [57].

In the present study, a range of broad specificity transporters, including inorganic/organic cation/anion, amino acid, oligopeptide, sugar and multivitamin transporters, are revealed to be enriched in *P. striatus* MTs, implying the specific function of the tubules in osmoregulation and organic solute transport. Based on the results of our study and previous studies of MTs of *Drosophila melanogaster* (Diptera) [25] and *Trichoplusia ni* (Lepidoptera) [26], we schematically illustrated the molecules associated with transepithelial fluid transport in *P. striatus* MTs (Fig. 4), in which the location of proteins in the hemolymph side and the tubule lumen side are derived from the two previous studies.

We revealed that the Kir channel, chloride channel and the sodium/hydrogen exchanger were highly expressed in MT4 (Fig. 4D; Additional file 9: Fig. S6). This indicates that MT4 is the main site for the transportation of K^+^, Cl^−^ and Na^+^. MT1+2 is also the main site for the transportation of K^+^, Cl^−^ and Na^+^, since MT1+2 expressed sodium-(potassium)-chloride cotransporter and sodium/potassium-transporting ATPase at levels similar to those in MT4. MT1+2 is also the main site for the absorption of water and emission of CO_2_ on account of the high expression of AQPs and carbonic anhydrases (Fig. 4B; Additional file 9: Fig. S6), respectively. An investigation of the leaf beetle *Phyllotreta armoraciae* showed that sugar transporters expressed in the tubules enable MTs to reabsorb and accumulate plant defense compounds (e.g., glucosinolate) from the tubule lumen to prevent the excretion of such substances for defense against predators [58]. Hence, we presume that the high expression of sugar transporters in MT1+2 and MT4 may confer *P. striatus* MTs the ability to defend against predators. In addition, zinc transporter and calcium transporter were enriched in MT3 (Fig. 4C; Additional file 9: Fig. S6), indicating that this segment may be mainly involved in the sequestration and excretion of multiple metals. This is in line with the results of related previous studies [59–63].

### Detoxification in MTs

Phytophagous insects feed on plant tissue, xylem/phloem fluids, nectar or pollen which are exposed to xenobiotics (i.e., insecticides, fungicides, antibiotics or noxious plant secondary metabolites, etc.). When confronted with toxic chemical compounds presented in the diet or produced by the metabolism of themselves, insects can successfully survive through their detoxification systems. Previous studies revealed that the salivary glands and alimentary tract of vector leafhoppers have the ability in the metabolism of natural and synthetic xenobiotics/toxins by expressing a variety of detoxification enzymes [51–53]. Previous studies also suggest that MTs of phytophagous insects display the abundance of enzymes related to detoxification of plant defense compounds, insecticides and other xenobiotic compounds [3, 5, 8, 10, 21, 22, 46, 49, 50, 64], making the tubules a major site of insecticide detoxification and resistance [49, 65, 66].

The differential expression and regionalized enrichment of detoxification-related unigenes among different MT regions of *P. striatus* indicate that these regions are separately specialized for targeting specific substrates to improve the detoxification efficiency and to accommodate rapid metabolic processes as the fluid passes through the tubules (Fig. 5; Additional file 10: Table S4). For example, GSTs that were highly expressed in MT1+2 and MT4 when compared with MT3 are associated with resistance to all major classes of insecticides. UGTs that were highly expressed in MT1+2 and MT4 when compared with MT3 catalyze the conjugation of sugar with small lipophilic substrates that need to be eliminated [67]. Catalases that were exclusively highly expressed in MT1+2 when compared with MT3 and MT4 catalyze the conversion of H_2_O_2_ to water and oxygen to prevent poisoning of cells [65]. In addition, MRPs that were exclusively highly expressed in MT1+2 when compared with MT3 and MT4 are a class of the ABCC subfamily that can confer drug resistance [68]. In insects, ABCC members are thought to be involved in response to dietary exposure to xenobiotics/toxins, which facilitate xenobiotic/toxins excretion and clearance [68]. Our results indicate that MT1+2 and MT4 play more important roles in biodegradation and metabolism of endogenous secondary metabolites and xenobiotics/toxins when compared with MT3, and the detoxification function derived from the tubules together with the salivary glands and alimentary tract might allow *P. striatus* to survive on a variety of host plants (e.g., winter wheat, corn and green foxtail, etc. [29]) and thus over summer. The relatively wide host range of this vector leafhopper species may indirectly help the transmission and spread of phytoplasma which triggers WBD disease.

### Immunity in MTs

**Fig. 7:**
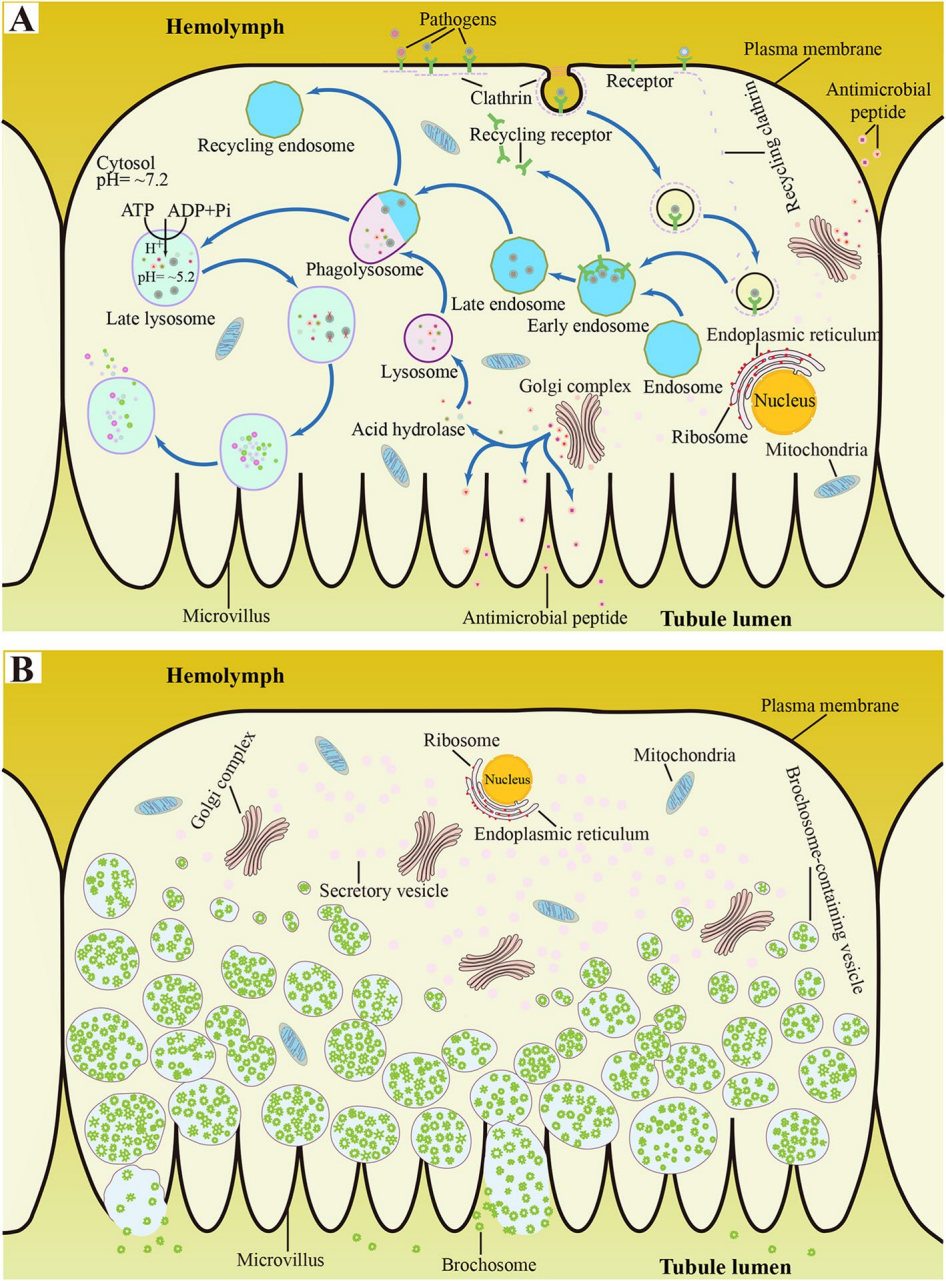
Schematic illustrations of the mechanisms of resistance to the invading pathogens in different MT regions of *P. striatus*. **A** The process of immunological responses speculated (including cellular immunity and humoral immunity) in a cell of MT1+2 and MT4. **B** A secretory cell of MT3 secreting brochosomes

Unlike vertebrates which have adaptive immunity, insects activate innate immune reactions when pathogens breach physical barriers (e.g., external cuticle, gut or trachea) and chemical barriers (e.g., low pH and antimicrobial factors such as lysozyme in the gut) to enter hemolymph, including cellular immunity and humoral immunity [69]. The alimentary tract and salivary glands of vector leafhoppers have been shown to attribute to insect immunological response in the resistance or reduction of microbial infection [53, 54]. Research during the past several decades on insect MTs has uncovered that the tubules contribute to activating innate immune defense to withstand the invading pathogens [3–5, 8, 46, 70]. In the present study, we revealed that lysosomal-related acid hydrolases (e.g., proteases, glycosidases and sulfatases) and integral membrane proteins (e.g., lysosomal-associated transmembrane proteins and lysosome-associated membrane glycoprotein) were highly expressed in MT1+2 and MT4 (Additional file 11: Table S5; Additional file 12: Figure S7), which are known or speculated to be involved in cellular responses to inflammation and pathogenic infection [43]. Coupled with the observations that a lot of mitochondria, secretory vesicles and extensive rough endoplasmic reticulum existed in MT1+2 and MT4 [32], our findings indicate that *P. striatus* MTs, especially the segments MT1+2 and MT4, can produce lysosomal-related acid hydrolases to resist and kill the encountered pathogens by phagocytosis. We presume that during the process of phagocytosis, pathogens first interact with specific receptors before internalized via clathrin-mediated endocytosis, then they fused with lysosomes to form phagolysosomes, and ultimately hydrolytically digested under the action of acid hydrolases (Fig. 7A).

Besides the abovementioned proteins involved in cellular immunity that eliminate the invading pathogens, we also found humoral immunity-related unigenes which are involved in the removal of pathogens by inducing the production of AMPs in *P. striatus* MTs, including four classes of immune-recognition receptors (i.e., PGRP, *β*-GRP, integrin and SR) (Fig. 7A; Additional file 11: Table S5; Additional file 12: Fig. S7). PGRP, a Gram positive-binding protein that exhibits the affinity to peptidoglycan identified in the moth *Trichoplusia ni* [71], was significantly highly expressed in MT1+2 and MT4 when compare with MT3. This implies that MT1+2 and MT4 are related to recognizing Gram-positive bacteria. *β*-GRP, a Gram-negative binding protein binding to lipopolysaccharides and β-1,3-glucan [72], was exclusively highly expressed in MT4 when compare with the MT1+2 and MT3. Integrin, a cell surface receptor that comprises a family of structurally homologous glycoprotein complexes and plays an important role in the recognition of appropriate receptors [73], was significantly highly expressed in MT1+2 and MT4 when compare with the MT3. SR, a transmembrane glycoprotein on the cell surface that possesses the capability to recognize pathogen-associated molecular patterns and mediate phagocytosis and the production of AMPs in *Micropilits mediator* wasps [74], was exclusively highly expressed in MT4 when compared with the MT1+2 and MT3. In addition, three classes of effector molecules (defensin, SOD and Prx) were identified in *P. striatus* MTs (Fig. 6A; Additional file 11: Table S5; Additional file 12: Fig. S7), which are associated with immune response in vertebrates and invertebrates [75]. For example, defensin, a family of endogenous cationic AMPs [76], has also been identified in the vector mosquito *Aedes aegypti* [77] for the degradation of ingested bacteria. SOD can convert •O^2−^ (produced in insect immune responses for killing microorganisms) into hydrogen peroxide (H_2_O_2_) which is less toxic and is subsequently removed from the cell to prevent H_2_O_2_ poisoning via conversion into water and oxygen by catalase [65] and Prx [78].

The above findings argue that MT1+2 and MT4 of *P. striatus* are the main sites to resist pathogens that have entered the hemolymph, and that MT1+2 and MT4 have different strategies to recognize and eliminate different types of pathogenic microorganisms (Fig. 7A). However, MT3, the remarkably inflated segment, may function to resist the invasion of pathogens in form of the secretion of protective material on the integument (Fig. 7B, see below).

### Secretion of protective material in MTs

Brochosomes are hollow honeycomb secretory granules (usually 0.2–0.7 μm in diameter) produced intracellularly and matured inside the Golgi complex in most of the cytoplasm (Fig. 7B) from specialized glandular/inflated MT segment (i.e., MT3) in leafhoppers and treehoppers [35]. Brochosomes are released and applied onto the integument and eggs via anointing by the hind legs of the insect [15]. Following anointing and grooming, brochosomes are spread evenly and often seal intersegmental folds which serve as the access of penetration for pathogens. Consequently, a dense layer of integument brochosomes (Fig. 1) function as a mechanical barrier, which may keep the attached spores far enough from the cuticle to prevent their germination on account the spores known to infect leafhoppers are 3–100 times larger than the brochosomes found in most leafhoppers [79]. Furthermore, bedsides resisting pathogens, the superhydrophobic brochosome coatings also protect the insect from rain or dew water, sticky liquid excretion and other environmental hazards (e.g., desiccation, temperature fluctuations, or solar radiation), predators, and parasites [15–18, 80, 81].

A study of the leafhopper *Graphocephala fennahi* indicates that brochosomes are mainly composed of a novel family of 21–40 kDa secretory proteins which are cross-linked by disulfide bonds [82]. These proteins have no homologies with the known proteins, which exhibit the characteristics of taxonomically restricted genes (also called orphans, encoding important taxon-specific traits) that are restricted to the superfamily Membracoidea [82]. Protein disulfide-isomerase located in the rough endoplasmic reticulum catalyzes the formation and rearrangement of intrachain and/or interchain disulfide bonds during protein folding, which has the function in stabilizing protein structures [83]. Protein disulfide-isomerase is the 94th most expressed gene in MTs of *G. fennahi*, and is speculated to be related to the durability of brochosomes [82]. Acidic ribosomal protein P located in the active site of the ribosome is the main component of the ribosome, which can improve the efficiency and accuracy of protein biosynthesis [84]. In our present study, protein disulfide-isomerase and acidic ribosomal protein P are in the list of the top 100 most abundant expressed unigenes in MT3 of *P. striatus* (Additional file 5: Table S2). Combined with the results of ultrastructural observations on MTs of various leafhopper species [32, 35], we presume that disulfide-isomerase and acidic ribosomal protein P are closely correlated to the biosynthesis of brochosomes in MT3. Moreover, a bulk of unigenes that were highly expressed in MT3 are novel, which may also be related to the biosynthesis of brochosomes. Some of these unigenes are probably taxonomically restricted genes, and thus can be attractive candidate genes for future studies of the biosynthesis and the origin of brochosomes in Membracoidea.

*Psammotettix striatus* is currently the only known vector leafhopper species that transmit phytoplasma triggering the WBD disease. Other insects with piercing-sucking mouthparts, such as *Tettigella viridis*, *Aphis gossypii*, *Macrosiphum avenae*, *Myzus persicae*, *Rhopalosiphum padi*, *R. maidis* and *Schizaphis graminum*, coexist with *P. striatus* in wheat fields where the WBD was prevalent during the wheat-growing season, but they fail to transmit phytoplasmas [27]. As one of the plant phloem-feeders, *P. striatus* acquire and transmit the phytoplasma by feeding on the sap in a non-destructive manner [27], since phytoplasmas are phloem-limited [36]. Phytoplasmas in vivo can pass through epithelial cells of the midgut to enter the hemolymph, where they may infect other tissues such as the MTs [85], fat bodies and brain [86, 87], or reproductive organs [88], then replicate in these tissues. To be transmitted to plants, phytoplasmas must penetrate specific cells of the salivary glands and must accumulate at a high level in the posterior acinar cells of the salivary glands before they can be transmitted [89]. At each point during this process, if the phytoplasmas fail to enter or exit the salivary glands, the insect would become a dead-end host and would be unable to transmit the phytoplasmas. Non-vector leafhoppers can be infected with a phytoplasma but yet be unable to transmit it to healthy plants [90, 91], perhaps because of the salivary gland barriers or other barriers. Previous studies of salivary glands and alimentary tract in vector leafhoppers [53, 54], coupled with the present study of MTs, suggest that these three organs are involved in the immune response. However, they have not blocked the flow and transmission of the phytoplasmas. This suggests that the phytoplasmas, the corresponding vector insects and their host plants may have formed an interdependent coevolutionary relationship.

Results of our present study indicate that MTs of leafhoppers have the ability to sense pathogenic challenges and mount effective killing responses by triggering the generation of effector activities (phagocytosis) and effector molecules. Prediction of physiological function in *P. striatus* MTs based on transcriptome gives clues to elucidating the tripartite phytoplasma-vector-plant interactions. Although whether non-vector leafhopper species that do not transmit the phytoplasma related to WBD disease is due to their immune response of the MTs or other organs such as the salivary glands and/or the alimentary tract remains unknown. Further studies of the function of MTs of more leafhoppers are hopeful to provide a theoretical basis for exploring phytoplasma transmission in vector species. In addition, we revealed that the Kir channel was highly expressed in MT4 of *P. striatus*, which plays a prominent role in K^+^ transportation [65] and has been verified as a target of selective insecticide flonicamid for the control of Hemipteran and Lepidopteran pests [66]. Similarly, some key sites or molecules participating in the execution of the normal physiological function of the tubules, such as AQPs highly expressed in MT1+2 of *P. striatus*, are important for the survival of plant-sap feeders [67, 92]. They will become attractive candidate target sites to be excavated for developing effective pest control agents.

## Conclusions

This study establishes a de novo transcriptome of *P. striatus* MTs. Results of our analyses indicate that MTs of leafhoppers perform multiple physiological functions as versatile organs than just excretory organs with osmoregulatory function, and that different MT regions show heterogeneity of physiological functions. The functions in terms of osmoregulation, organic solute transport, detoxification and immunity may allow *P. striatus* to resist pathogens from habitats and to utilize a wider range of host plants, which may assist the transmission and oversummer of phytoplasmas. Disulfide-isomerase, acidic ribosomal protein P and other unigenes that were highly expressed in MT3 involve in brochosome biosynthesis, which can be attractive candidate genes for future studies of the biosynthesis and the origin of brochosomes.

## Methods

### Sample preparation

All leafhoppers of *P. striatus* were obtained from a field collection in Shaanxi Province, China during its emergence period of 2020. The leafhoppers were maintained at 25 ± 1 °C under relative humidity 50 ± 5% with a photoperiod of 14 h light/10 h dark, and reared on cultivated wheat seedlings in the laboratory of Northwest A&F University.

The adult leafhoppers of both sexes were used for the isolation of *P. striatus* MTs in this study. Briefly, each individual was frozen anesthetized for 3 min at 4 °C, and then the dissection was performed on ice-cold sterile phosphate buffer solution (PBS, pH = 7.2) treated with o.1% diethylpyrocarbonate under an Olympus SZX 10 stereomicroscope (Olympus Corporation, Japan). MTs were detached from the alimentary tract, the parts of each tubule embedding the filter chamber and rectum not included, with fine pins and micro forceps, and rinsed by ice-cold sterile PBS three times to remove the impurities. Then the detached MTs were immediately transferred into ice-cold RNAlater™ Stabilization Solution (ThermoFisher Scientific, USA) and stored at − 80 °C for subsequent RNA extraction. To generate RNA pools ca. 1800 dissected adults were used for three biological replicates of the three regions, resulting in a total of nine cDNA libraries (3 regions × 3 replicated samples).

### RNA extraction, library construction and Illumina sequencing

Total RNA of all dissected tissue was extracted using Trizol Reagent Kit (Invitrogen, USA), according to the manufacturer’s protocol. The degradation and contamination of total RNA of each library were assessed on an Agilent 2100 Bioanalyzer (Agilent Technologies, USA) and checked using RNase-free 1% agarose gel electrophoresis. The purity, concentration and integrity of total RNA of each library were verified using the NanoPhotometer spectrophotometer (IMPLEN, USA), Qubit 2.0 Fluorometer (Life Technologies, USA) and Agilent Bioanalyzer 2100 system (Agilent Technologies, USA), respectively. High-quality RNA samples (OD260/280 = 1.8–2.2, OD260/230 ≥2.0, RIN ≥8.0) were used to construct sequencing library.

After total RNA was extracted, mRNA was isolated using Oligo (dT) magnetic beads. Then the isolated mRNA was fragmented into short fragments using fragmentation buffer and used as the template for reverse transcription to the first-strand complementary DNA (cDNA) synthesis with random primers. Second-strand cDNA was synthesized by using DNA polymerase I, RNase H, dNTP and buffer. Then, the double-strand cDNA (dsDNA) fragments were purified with QiaQuick PCR extraction kit (Qiagen, The Netherlands), end-repaired, poly (A) added, and ligated to Illumina sequencing adapters. The ligation products were size-selected by agarose gel electrophoresis, PCR amplified and sequenced using Illumina HiSeq™ 6000 by Gene Denovo Biotechnology Co., Ltd. (Guangzhou, China). RNA extraction and library preparation were performed for all samples in parallel to avoid any day or batch effects.

### Assembly, annotation and bioinformatic analysis

For acquired high-quality clean reads, raw reads in FASTQ format obtained from the sequencing machines were trimmed to remove Illumina adapters, reads containing more than 10% of unknown nucleotides, poly N and low-quality reads containing more than 50% of low quality (Q-value ≤20) bases by fastp v0.18.0 [93] with default parameters. The high-quality clean reads were assembled into unigenes using the short reads assembling program Trinity v2.8.4 [94] with default parameters. Meanwhile, Q20, Q30, GC-content and sequence duplication levels of the clean data were calculated to ensure the data quality. The integrality of sequence assembly was evaluated with the BUSCO v3 [95]. All the downstream analyses were based on clean data with high quality.

The longest transcript of each gene was defined as the “unigene” for functional annotation. To acquire the coding region and basic functional information, all unigenes were against the Nr, Swiss-Prot, KEGG [96], KOG and GO databases, using BLASTx program (http://www.ncbi.nlm.nih.gov/BLAST/) with an E-value threshold of 1.0E–5. Protein functional annotations and pathway annotations could then be obtained according to the alignment results of the best hit.

### Analysis of differentially expressed genes (DEGs)

The repeatability of experiment results and operational stability between libraries were evaluated by PCA and the calculated Pearson correlation coefficient of pairwise comparison. The level of each gene expression was estimated by calculated and normalized RPKM [97] using the average data of three biological replicates. According to the RPKM values, DEGs analysis among different regions was identified by DESeq2 [98] with the parameter of false discovery rate (FDR) below 0.05 and absolute fold change (|log_2_ FC| >1) as a threshold. Unigenes with RPKM values of less than five were filtered. Bioinformatic analysis was performed using Omicsmart tools, a dynamic and interactive online platform for data analysis (https://www.omicsmart.com). Hierarchical clustering heatmaps were produced using the OmicShare tools (Z-score normalization set to row, and all other parameters set as default) based on normalized RPKM data.

### Functional classification and metabolic pathway analysis

To recognize the main biological functions of DEGs, the GO terms functional classification of all identified DEGs and the gene number of the related GO terms were applied to statistically analyze the main function of unigenes according to Gene Ontology. To understand the gene’s biological function, metabolic pathway enrichment analysis was done by comparing the DEG number of the metabolic pathway to that of the background gene number with the KEGG pathway database (http://www.genome.jp/tools/kaas/). The calculated *p*-value was gone through FDR correction, taking FDR <0.05 as a threshold. GO terms/pathways meeting this condition were defined as significantly enriched GO terms/pathways in DEGs.

### Validation of RNA-Seq accuracy by RT-qPCR

Twelve DEGs were selected to confirm the reliability of RPKM analysis from RNA-Seq using RT-qPCR. RNA extraction for each sample was conducted as described above, reverse-transcribed to cDNA using PrimeScript RT reagent Kit (TaKaRa, Japan). RT-qPCR reactions were performed on the ABI 7500 Fast Real-Time PCR Detection System (Applied Biosystems, USA), with three independent technical replicates and three independent biological replicates for each gene. A 20 μl reaction mixture consisted of 2 × T5 Fast qPCR Mix (SYBR Green I) 10 μl, 0.8 μl each of the forward and reverse primers (10 μM), 1 μl of cDNA template, and 7.4 μl of RNase-free ddH2O. Negative controls without template (replaced by nuclease-free water) were included in the experiment to detect contamination and to determine the degree of dimer formation. RT-qPCR was conducted with standard thermal cycle procedure as follows: 95 °C for 1 min, followed by 40 cycles at 95 °C for 15 s, 60 °C for 15 s and 72 °C for 30 s. Following amplification, a melting curve analysis was performed to assess the specificity of the PCR product. Relative mRNA expression levels for each gene in each sample were calculated using the 2^–ΔΔC^_T_ method [99] with *Elongation factor 1α* (*EF 1-α*) and *60S ribosomal protein LP2* (*RPLP2*) as normalized internal reference genes. Relevant gene-specific primers for RT-qPCR were designed by Primer Premier v5.0 with the predicted coding sequences as reference sequences. Data analysis of relative expression levels of selected genes was compared with Student’s *t*-test between RT-qPCR and RNA-Seq using SPSS v23.0 (IBM Corporation, USA). Results are exhibited by mean ± standard error (SE), and each data point represents the average of three biological replicates. Significance and high significance were defined as *p*-value <0.05 and *p*-value <0.01, respectively.

## Supplementary Information

**Additional file 1: Table S1**. Statistics of the sequencing data from nine cDNA libraries of *P. striatus* MTs. (XLS 92 kb)

**Additional file 2: Figure S1**. Annotation of unigenes from *P. striatus* MTs. (A) Statistics of the results of unigenes annotated in five databases (Nr, KEGG, KOG, Swiss-Port and GO). (B) Annotated results of unigenes in the Nr database. (C) Annotated results of unigenes in the Swiss-Prot database.

**Additional file 3: Figure S2**. Functional classification of unigenes from *P. striatus* MTs. (A) Gene Ontology classification of assembled unigenes. The X-axis is the GO term of GO categories, and the Y-axis is the number of genes annotated to the term. (B) KEGG pathway distributions of assembled unigenes. The X-axis is the number of genes annotated to the pathway. The Y-axis indicates the name of the KEGG metabolic pathway. (C) KOG functional classification of assembled unigenes. The X-axis is the name of 25 groups of KOG. Y-axis indicates the number of unigenes annotated to the group. Each category is indicated on X-axis by a letter listed in the column on right.

**Additional file 4: Figure S3**. Maps of Pearson correlation coefficient in pairwise comparison among replicates for transcriptome sequencing quality analysis.

**Additional file 5: Table S2**. Top 100 abundant unigenes in each region of *P. striatus* MTs. (XLS 128 kb)

**Additional file 6: Figure S4**. KEGG enrichment analysis of DEGs in pairwise comparison among different MT regions of *P. striatus*. Gene ratio refers to the ratio of the number of DEGs in the pathway to the total number of unigenes that are located in the pathway. The larger the gene ratio, the higher the degree of enrichment is. The dot size indicates the number of DEGs of the pathway, and the dot color indicates the *p*-value (the same below).

**Additional file 7: Figure S5**. GO enrichment analysis of DEGs in pairwise comparison among different MT regions of *P. striatus*.

**Additional file 8: Table S3**. Candidate DEGs related to osmoregulation and organic solute transport identified in *P. striatus* MTs. (XLS 39 kb)

**Additional file 9: Figure S6**. Normalized heatmap based on RPKM values of DEGs related to osmoregulation and organic solute transport of *P. striatus* MTs. Orange color indicates up-regulated expression, whereas blue color indicates down-regulated expression (the same below).

**Additional file 10: Table S4**. Candidate DEGs related to detoxification identified in *P. striatus* MTs. (XLS 48 kb)

**Additional file 11: Table S5**. Candidate DEGs related to immunity identified in *P. striatus* MTs. (XLS 51 kb)

**Additional file 12: Figure S7**. Normalized heatmap based on RPKM values of DEGs related to immunity of *P. striatus* MTs.

**Additional file 13: Table S6**. Specific primer pairs used in RT-qPCR. (XLS 369 kb)

## Data Availability

The sequence data (PRJNA743281) is accessible with the following link: https://www.ncbi.nlm.nih.gov/sra/PRJNA743281.

## Abbreviations

ABC transporters: ATP-binding cassette transporters
ADHs: Alcohol dehydrogenases
AMPs: Antimicrobial peptides
AQPs: Aquaporins
BUSCO: Benchmarking Universal Single-Copy Orthologs
DEGs: Differentially expressed genes
FDR: False discovery rate
GO: Gene Ontology
GSTs: Glutathione *S*-transferases
KEGG: Kyoto Encyclopedia of Genes and Genomes
KOG: euKaryotic Orthologues Group
MT1: The anterior segment
MT1+2: The anterior segment together with the sub-anterior segment
MT2: The sub-anterior segment
MT3: The inflated segment
MT4: The distal segment
MTs: Malpighian tubules
Nr: NCBI Non-redundant protein database
P450s: Cytochrome P450 monooxygenases
PBS: Phosphate buffer solution
PCA: Principal component analysis
RPKM: Reads Per Kilobase per Million mapped read
RT-qPCR: Reverse transcription-quantitative PCR
SODs: Superoxide dismutases
SRA database: Short Read Archive database
UGTs: Uridine diphosphate-glycosyltransferases
