# Supplementary figures and images for: Gene expression profiles in Malpighian tubules of the vector leafhopper Psammotettix striatus (L.) revealed regional functional diversity and heterogeneity
Source: BMC Genomics. 2022 Jan 21;23:67. doi: 10.1186/s12864-022-08300-6 (PMC8781387; doi:10.1186/s12864-022-08300-6)

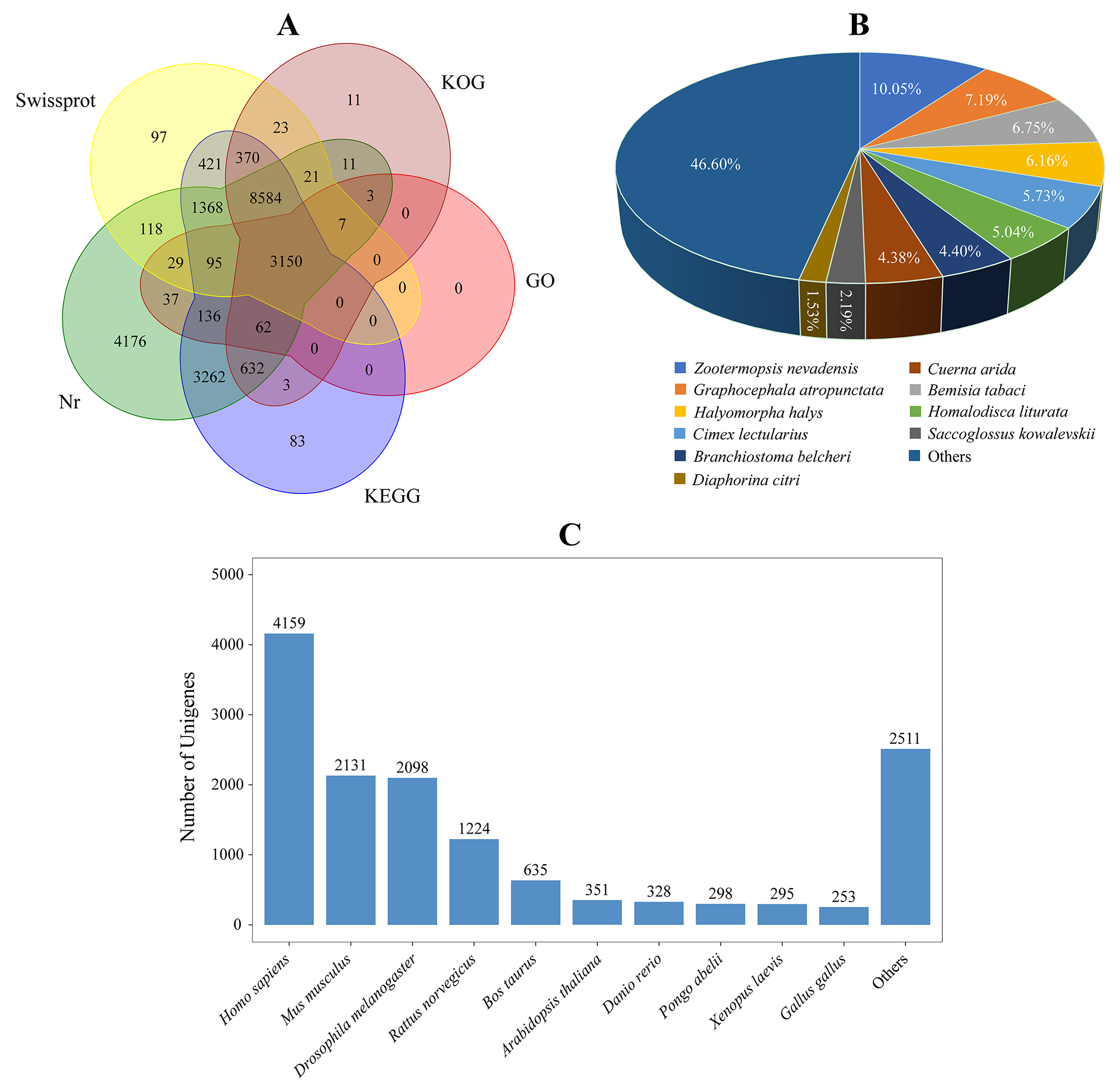

Supplement: Supplementary file 2 — Additional file 2: Figure S1. Annotation of unigenes from P. striatus MTs. (A) Statistics of the results of unigenes annotated in five databases (Nr, KEGG, KOG, Swiss-Port and GO). (B) Annotated results of unigenes in the Nr database. (C) Annotated results of unigenes in the Swiss-Prot database. [file 12864_2022_8300_MOESM2_ESM.tif]

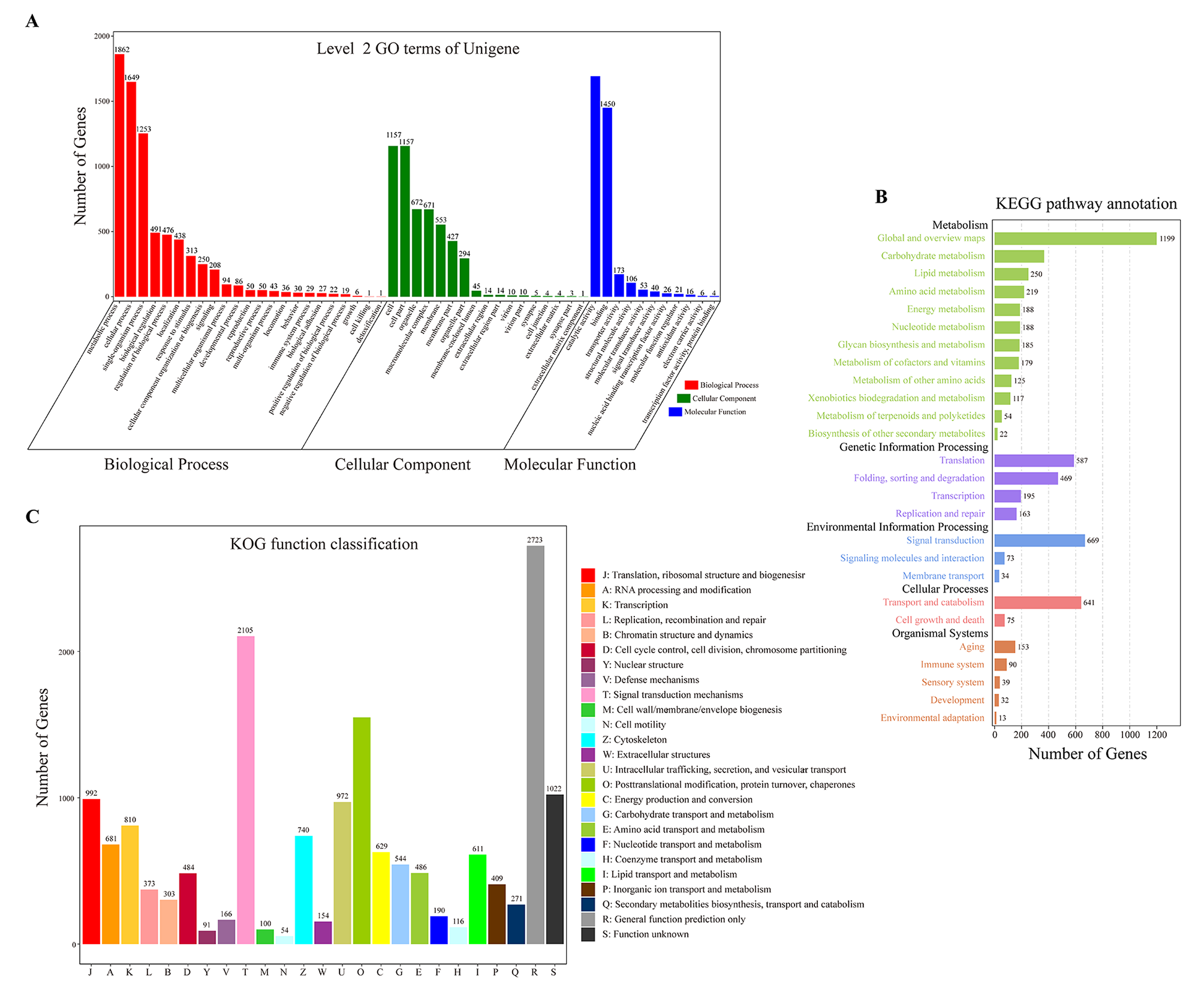

Supplement: Supplementary file 3 — Additional file 3: Figure S2. Functional classification of unigenes from P. striatus MTs. (A) Gene Ontology classification of assembled unigenes. The X-axis is the GO term of GO categories, and the Y-axis is the number of genes annotated to the term. (B) KEGG pathway distributions of assembled unigenes. The X-axis is the number of genes annotated to the pathway. The Y-axis indicates the name of the KEGG metabolic pathway. (C) KOG functional classification of assembled unigenes. The X-axis is the name of 25 groups of KOG. Y-axis indicates the number of unigenes annotated to the group. Each category is indicated on X-axis by a letter listed in the column on right. [file 12864_2022_8300_MOESM3_ESM.tif]

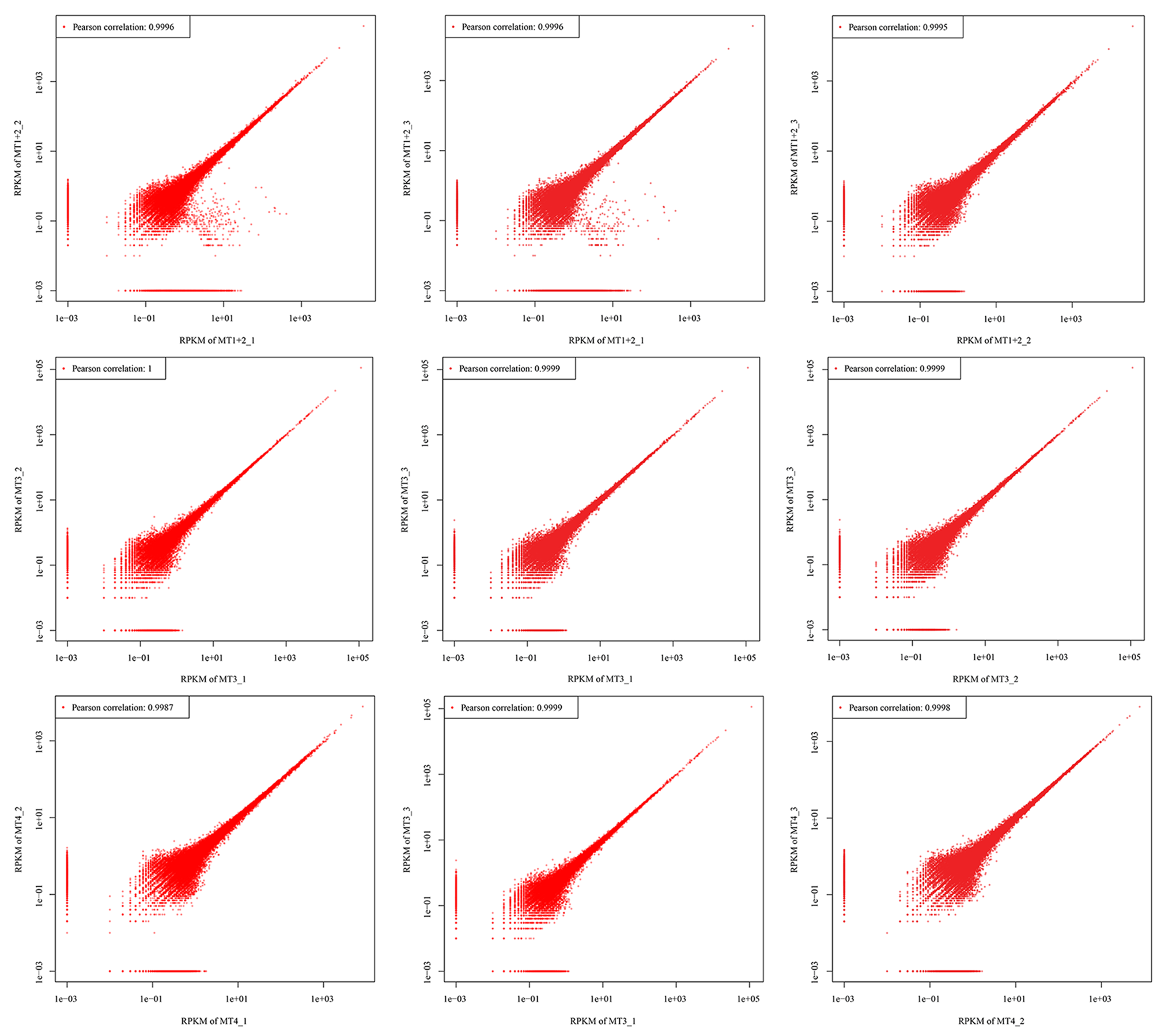

Supplement: Supplementary file 4 — Additional file 4: Figure S3. Maps of Pearson correlation coefficient in pairwise comparison among replicates for transcriptome sequencing quality analysis. [file 12864_2022_8300_MOESM4_ESM.tif]

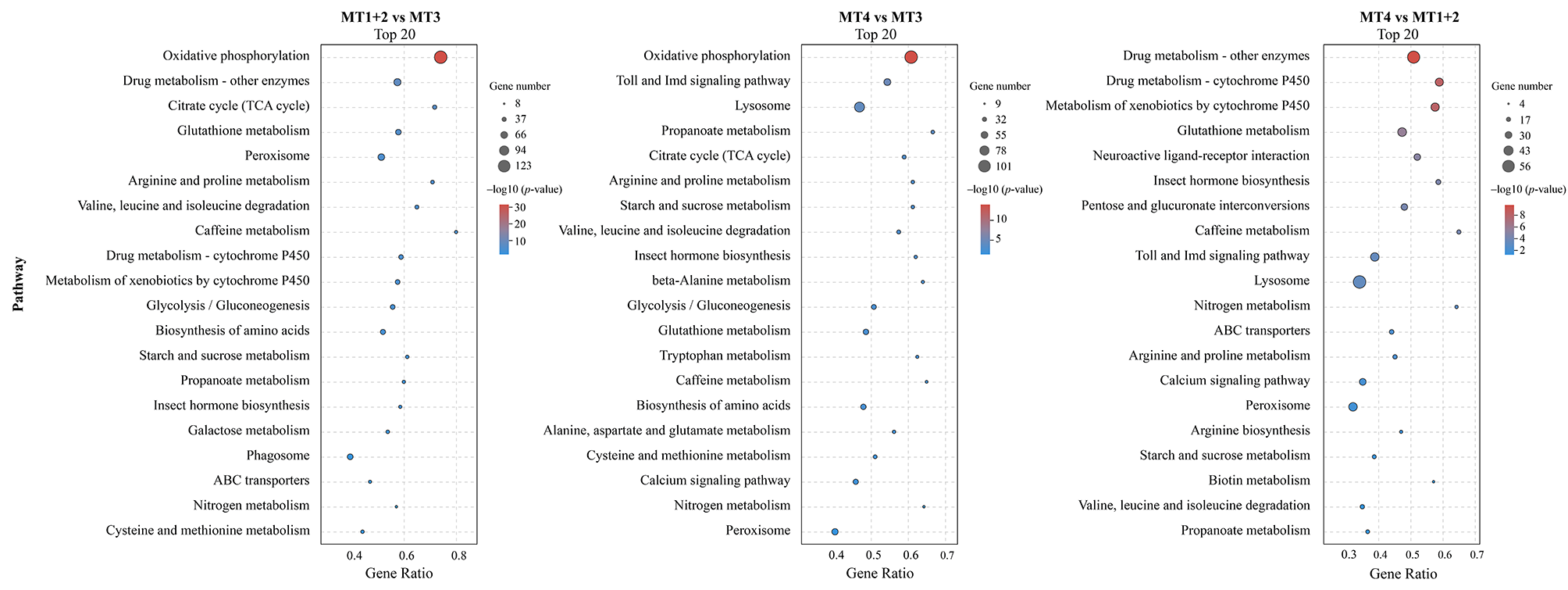

Supplement: Supplementary file 6 — Additional file 6: Figure S4. KEGG enrichment analysis of DEGs in pairwise comparison among different MT regions of P. striatus. Gene ratio refers to the ratio of the number of DEGs in the pathway to the total number of unigenes that are located in the pathway. The larger the gene ratio, the higher the degree of enrichment is. The dot size indicates the number of DEGs of the pathway, and the dot color indicates the p-value (the same below). [file 12864_2022_8300_MOESM6_ESM.tif]

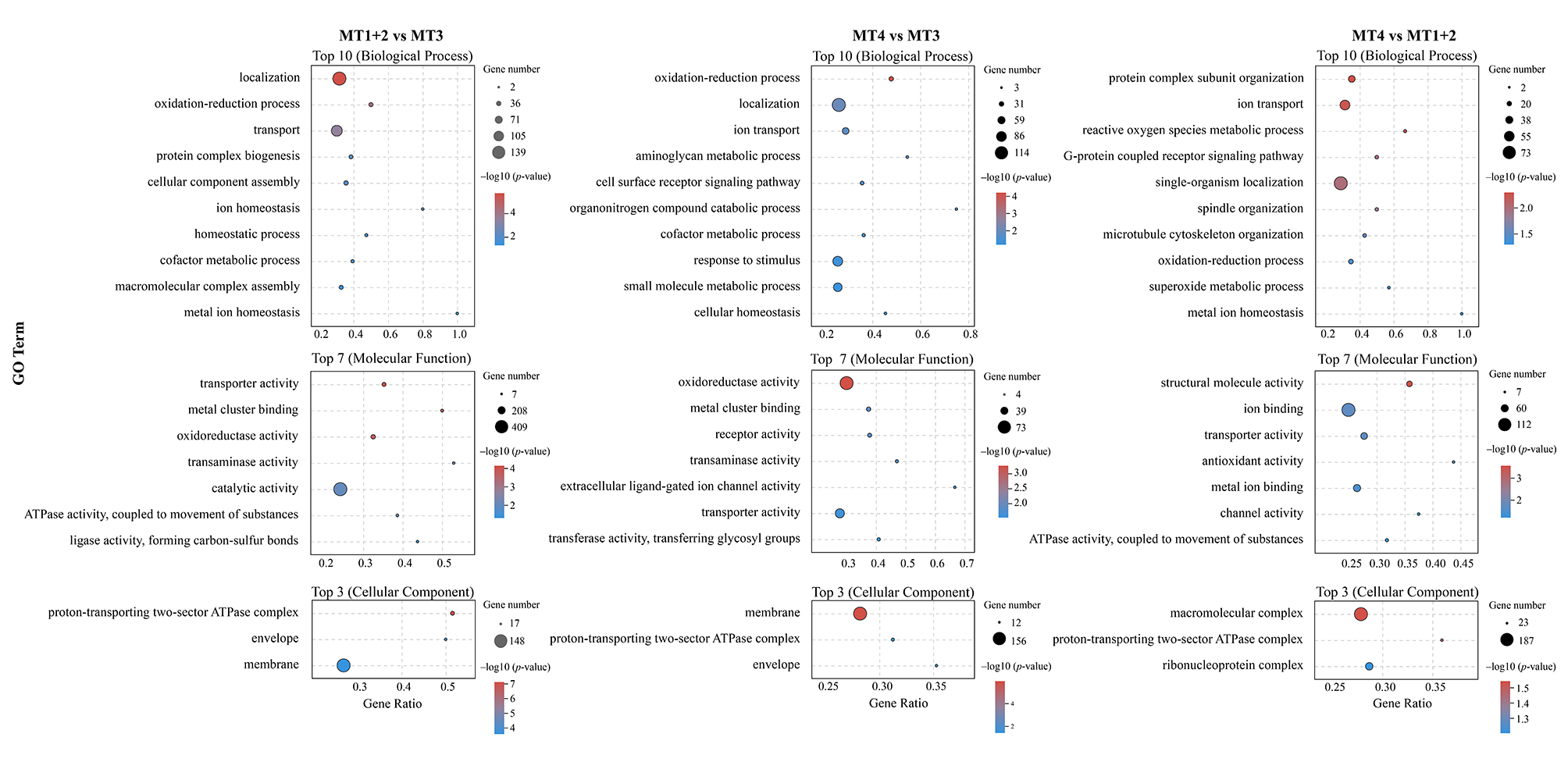

Supplement: Supplementary file 7 — Additional file 7: Figure S5. GO enrichment analysis of DEGs in pairwise comparison among different MT regions of P. striatus. [file 12864_2022_8300_MOESM7_ESM.tif]

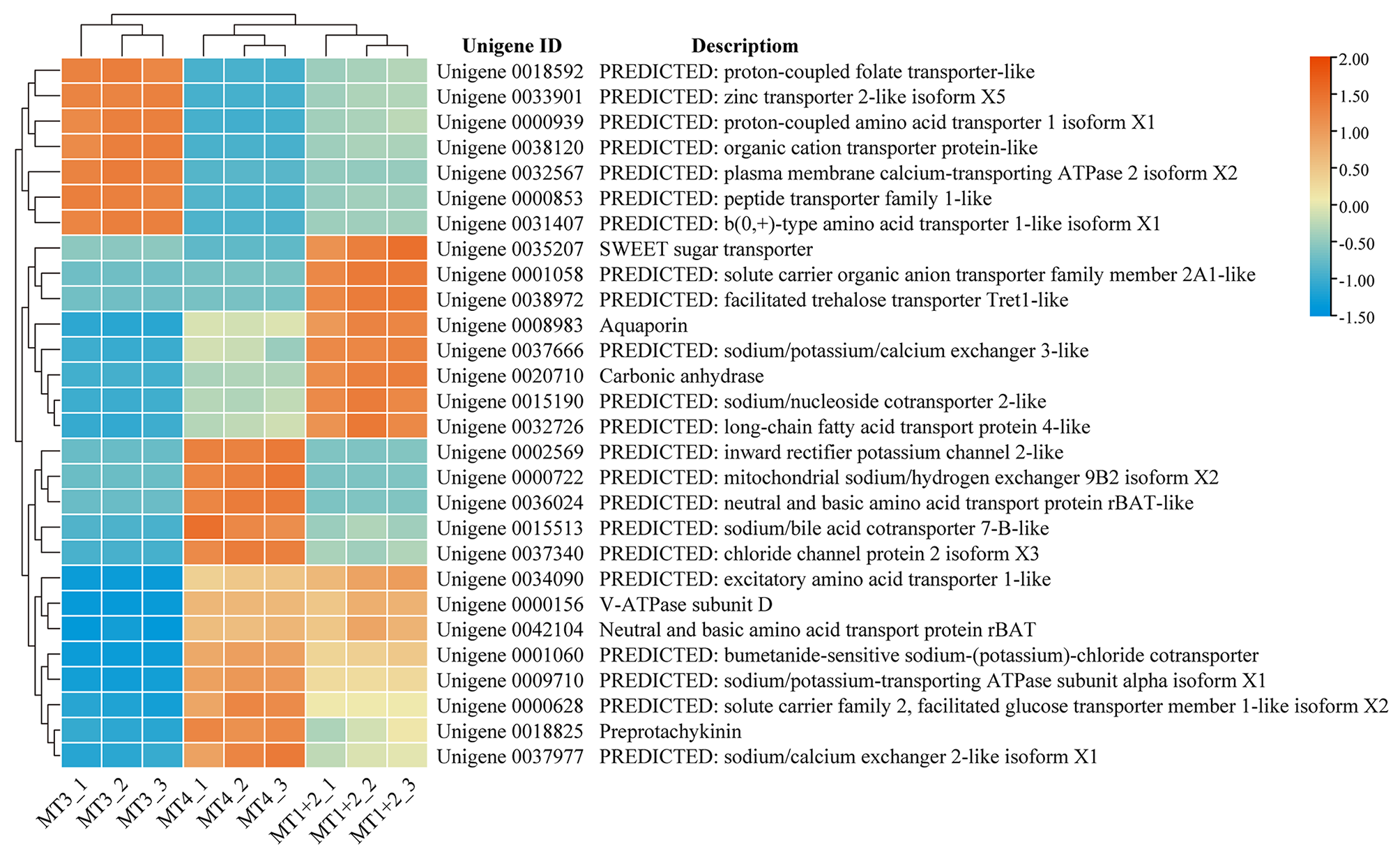

Supplement: Supplementary file 9 — Additional file 9: Figure S6. Normalized heatmap based on RPKM values of DEGs related to osmoregulation and organic solute transport of P. striatus MTs. Orange color indicates up-regulated expression, whereas blue color indicates down-regulated expression (the same below). [file 12864_2022_8300_MOESM9_ESM.tif]

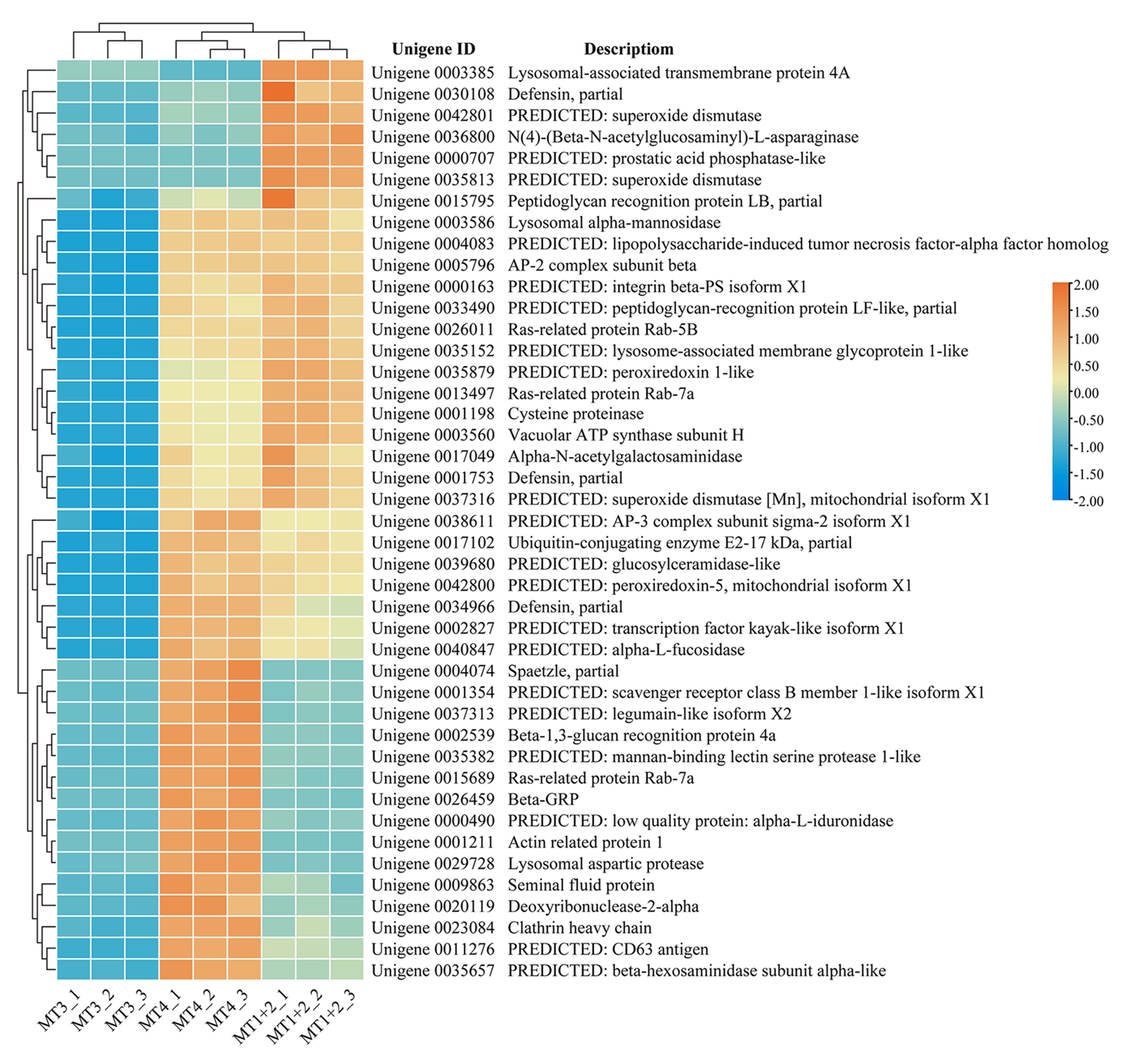

Supplement: Supplementary file 12 — Additional file 12: Figure S7. Normalized heatmap based on RPKM values of DEGs related to immunity of P. striatus MTs. [file 12864_2022_8300_MOESM12_ESM.tif]
